# Estimation of silent phenotypes of calf antibiotic dysbiosis

**DOI:** 10.1038/s41598-023-33444-0

**Published:** 2023-04-19

**Authors:** Shunnosuke Okada, Yudai Inabu, Hirokuni Miyamoto, Kenta Suzuki, Tamotsu Kato, Atsushi Kurotani, Yutaka Taguchi, Ryoichi Fujino, Yuji Shiotsuka, Tetsuji Etoh, Naoko Tsuji, Makiko Matsuura, Arisa Tsuboi, Akira Saito, Hiroshi Masuya, Jun Kikuchi, Yuya Nagasawa, Aya Hirose, Tomohito Hayashi, Hiroshi Ohno, Hideyuki Takahashi

**Affiliations:** 1 Kuju Agricultural Research Center, Graduate School of Agriculture, Kyushu University, Oita, 878-0201 Japan; 2 Graduate School of Horticulture, Chiba University, Matsudo, 271-8501 Japan; 3 RIKEN Integrated Medical Science Center, Yokohama, Kanagawa 230-0045 Japan; 4 Japan Eco-Science (Nikkan Kagaku) Co., Ltd., Chiba, 260-0034 Japan; 5 Sermas, Co., Ltd., Chiba, 271-8501 Japan; 6 RIKEN BioResource Research Center, Ibaraki 305-0074 Tsukuba, Japan; 7 RIKEN Center for Sustainable Resource Science, Yokohama, Kanagawa 230-0045 Japan; 8 Research Center for Agricultural Information Technology, National Agriculture and Food Research Organization, Tsukuba, Ibaraki 305-0856 Japan; 9 Feed‐Livestock and Guidance Department, Dairy Technology Research Institute, The National Federation of Dairy Co‐operative Associations (ZEN‐RAKU‐REN), Fukushima, 969-0223 Japan; 10 Pathology and Production Disease Group, Division of Hygiene Management, Hokkaido Research Station, National Institute of Animal Health, National Agriculture and Food Research Organization, Hokkaido, 062-0045 Japan

**Keywords:** Microbiology, Physiology

## Abstract

Reducing antibiotic usage among livestock animals to prevent antimicrobial resistance has become an urgent issue worldwide. This study evaluated the effects of administering chlortetracycline (CTC), a versatile antibacterial agent, on the performance, blood components, fecal microbiota, and organic acid concentrations of calves. Japanese Black calves were fed with milk replacers containing CTC at 10 g/kg (CON group) or 0 g/kg (EXP group). Growth performance was not affected by CTC administration. However, CTC administration altered the correlation between fecal organic acids and bacterial genera. Machine learning (ML) methods such as association analysis, linear discriminant analysis, and energy landscape analysis revealed that CTC administration affected populations of various types of fecal bacteria. Interestingly, the abundance of several methane-producing bacteria at 60 days of age was high in the CON group, and the abundance of *Lachnospiraceae*, a butyrate-producing bacterium, was high in the EXP group. Furthermore, statistical causal inference based on ML data estimated that CTC treatment affected the entire intestinal environment, potentially suppressing butyrate production, which may be attributed to methanogens in feces. Thus, these observations highlight the multiple harmful impacts of antibiotics on the intestinal health of calves and the potential production of greenhouse gases by calves.

## Introduction

The beneficial effects of administering antibiotics at nontherapeutic concentrations in feed as growth promoters (termed antimicrobial growth promoters, AGPs) were first recognized in the 1940s when chickens fed with streptomycin exhibited improved growth and feed efficiency^1^. Since then, the growth-promoting effects of several antimicrobial agents have been documented in cattle and other ruminants, poultry, and swine^2^. In particular, chlortetracycline (CTC), an antibacterial agent widely used in the livestock industry^3^, has the capacity of stimulating growth, reducing the incidence of calf scours, and improving feed efficiency^4^. In recent years, however, the use of AGPs has been under scrutiny from public health, food safety, and regulatory perspectives due to concerns about their potential to promote antimicrobial resistance (AMR)^5,6^. However, in the modern livestock industry, which depends on AGPs, it is unknown about how removing antibiotics from the diets of calves would affect their growth performance, nutrient metabolism, and intestinal environment.

The gut microbiota shapes key aspects of postnatal life, such as the development of the immune system^7,8^, and influences the host’s physiology, including the energy balance. In addition, antibiotic usage affects the gut microbiota profiles in humans^9^ and swine^10^. In broiler chickens, AGP administration alters the gut microbiota populations, which improves metabolism, the utilization of dietary carbohydrates and lipids, and energy harvest^11^. In addition, a previous study in mice found that AGPs directly affect host physiology and the gut microbiota, and this effect is associated with the promotion of growth^12^. However, the effects of antibiotics on the gut microbiota, characterized by the use of a culture-independent metagenomic approach, and host physiology have not been extensively studied in calves. Therefore, this study aimed to evaluate the effects of a lack of AGP administration on the growth performance, health conditions, and preweaning microbial diversity of beef calves. To comprehensively analyze the calf physiology, immunology, and the intestinal environment in response to feeding with and without AGP administration, procedures combining machine learning (ML) and statistical causal inference were used in the present study.

Here, the physiological properties and bacterial populations of calves with and without antibiotic treatment were evaluated in detail. Given that the differences between the two groups were difficult to discern through general statistical comparisons, the data were evaluated with a combination of ML and statistical causal inference to detect the potential treatment effects. These calculations were used to estimate the effect of antibiotics on symbiotic bacterial groups and the production of butyrate and methane. These observations provide critical information for the development of sustainable livestock technologies to protect animal health and the global environment.

## Results

### Outline of the study

**Figure 1:**
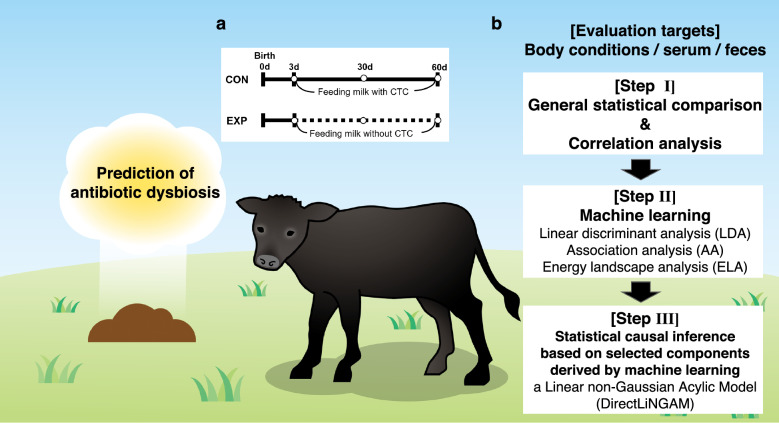
Experimental design, experimental procedure, and research objectives. (**a**) Illustration of the experimental design. In the early postnatal period [from day (**d**) 3 to d 60], when the rumen had not yet developed, the antibiotic treatment group (CON) and the nontreatment group (EXP) were established. (**b**) The analysis target and its procedure are shown. In Step I, general statistical comparisons and correlation analyses were performed; in Step II, to screen for potential relationships among factors, supervised linear discriminant analysis (LDA), unsupervised association analysis (AA), and energy landscape analysis (ELA) were performed as machine learning (ML). In Step III, to statistically determine the causal relationship between the factors narrowed down by ML, linear non-Gaussian acyclic model (DirectLiNGAM) analysis, which can be applied to non-Gaussian distributions, was implemented to statistically estimate the causal relationship between factors refined by ML. These three steps allowed us to infer the mechanism of intestinal disturbance induced by antibiotics (dysbiosis) in calves.

To investigate how lack of AGP administration in the preweaning period affects the growth performance, health condition, and microbial diversity, calves were fed with milk replacer containing CTC, an antibacterial agent widely used in the livestock industry, at 10 g/kg (CON group) or 0 g/kg (EXP group) (Fig. 1a). The data were analyzed in three steps as described in Fig. 1b. In the first step (Step I), the data were analyzed by general statistical comparison and correlation analysis. Subsequently (Step II), to reveal the potential differences between the two groups, the data were further analyzed using ML methods, such as linear discriminant analysis (LDA), association analysis (AA), and energy landscape analysis (ELA). As a result, potential differences between treatments were identified. Then (Step III), to target the narrowed group of factors linked with the differences, we performed statistical causal inference using a linear non-Gaussian acyclic model (LiNGAM), which is easy to perform when variation is high. LiNGAM analysis can be performed to infer the causal relationship between two groups. Following these steps, the following studies are underway.

### General statistical comparison

**Figure 2:**
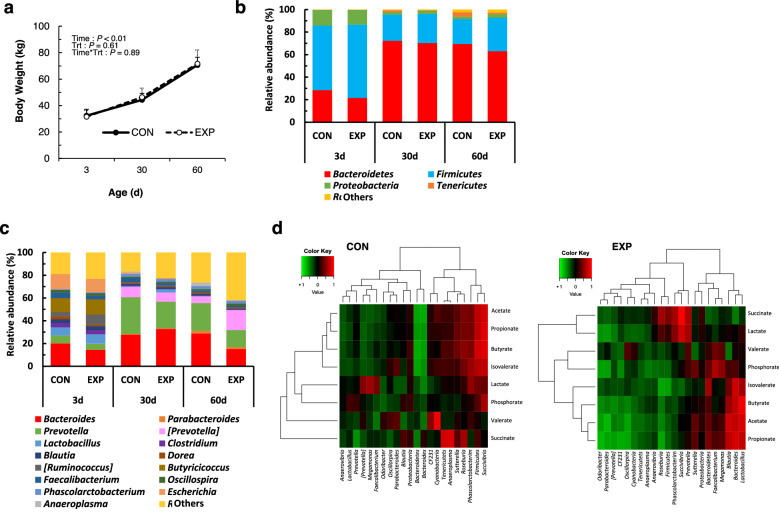
General statistical comparisons and correlation analyses. (**a**) Changes in BW during the period. Relative abundance of the fecal microbiota at the (**b**) phylum and (**c**) genus levels in calves fed milk replacer containing chlortetracycline (CTC) at 10 g/kg (CON) or 0 g/kg (EXP). (**d**) Correlation between fecal organic acids and bacterial genera during the period based on the sampling data at 30 and 60 d.

None of the calves developed major illnesses throughout the study. The administration of feed with or without AGPs to beef calves did not affect the calves phenotype, as indicated by no differences in the feed intake, body weight, or physical measurements (body height, body length, heart girth, and waist circumference) between the two groups (Figs. 2a and S1). The levels of serum components, cytokines, hormones, fecal short-chain fatty acids, and phosphate did not appear to differ significantly between the two groups (Figs. S2–S4). The abundances of the bacterial phyla and genera in the feces are shown in Fig. 2b and c, respectively. Regardless of the dietary treatments, the phylum Firmicutes was the most predominant at 3 days (d) of age, but the phylum Bacteroidetes became predominant at 30 and 60 d of age. At the genus level, either *Bacteroides* or *Prevotella* were predominant throughout the study. Differences in bacterial diversity and the major bacterial populations were also not always observed between the treatment groups (Figs. S5–S7). However, Adonis test revealed that the weighted UniFrac distances at 60 d of age were affected by CTC administration (*P* = 0.03) (Fig. S6a). β-diversity indices, including unweighted- and weighted Unifrac distances, changed with age in both treatment groups (*P* < 0.05) (Fig. S6b). Moreover, the correlations between the major fecal bacteria, organic acids, and phosphate were clearly different between the two groups (Figs. 2d and S8).

### ML results

**Figure 3:**
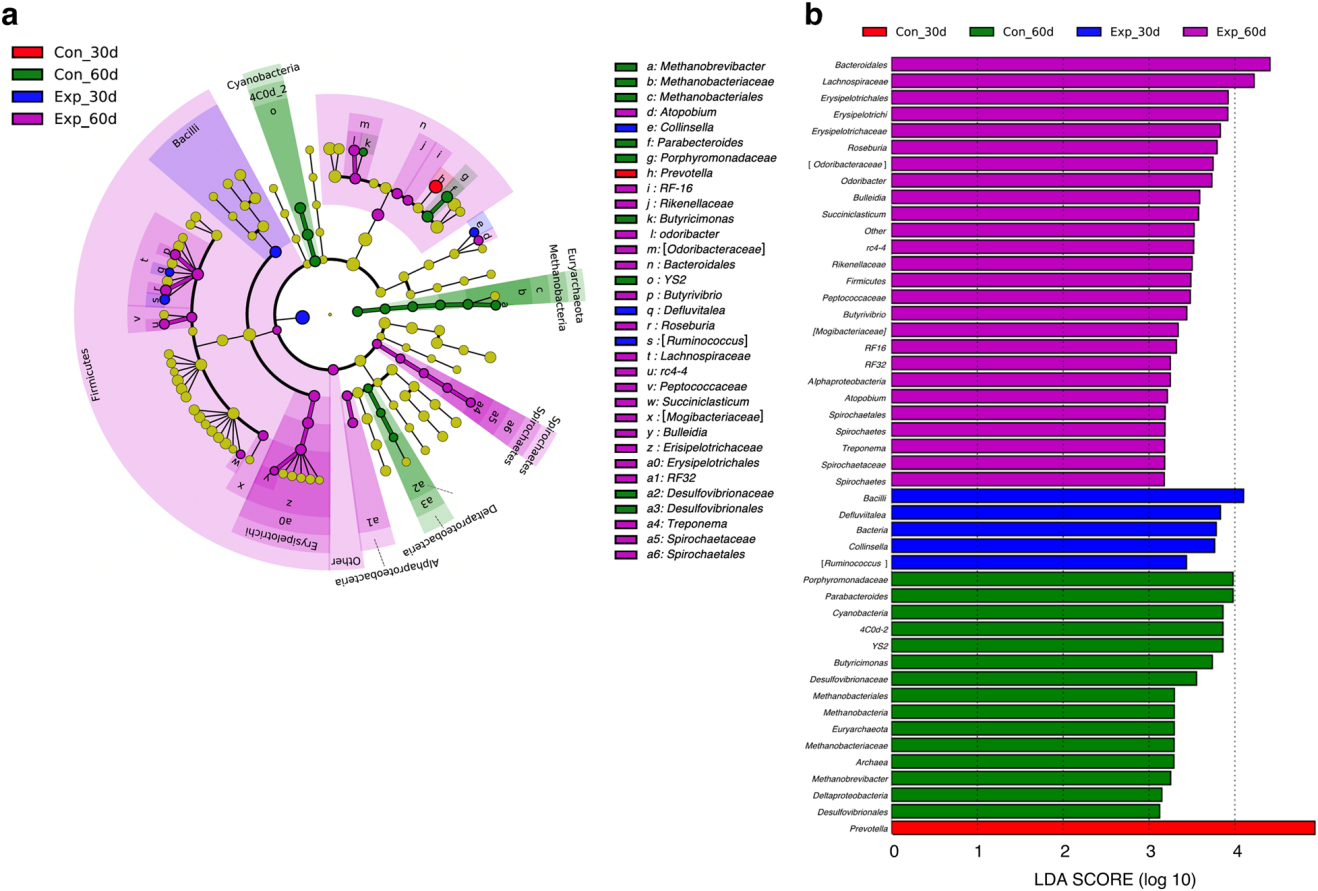
Screening for potential relationships among factors by linear discriminant analysis (LDA). (**a**) LDA effect size (LEfSe) cladogram visualized based on the significant changes in the bacterial population calculated by LDA (p < 0.05; > threefold change). (**b**) Significant changes in the bacterial population calculated by LDA (p < 0.05; > threefold change). CON group: group treated with antibiotics (n = 6); EXP group: group treated without antibiotics (n = 6).

LDA effect size (LEfSe) analysis, a supervised ML method, revealed that CTC treatment affected the populations of various types of fecal bacteria (Fig. 3). Interestingly, the abundance of the family *Lachnospiraceae* was higher in the EXP group at 60 d. In contrast, the abundance of methanogens, including class *Methanobacteria*, order *Methanobacteriales*, family *Methanobacteriaceae*, and genus *Methanobrevibacter*, was increased in the CON group at 60 d. In addition, the correlation between fecal bacterial abundance determined by the LDA and fecal levels of organic acid and phosphate was altered by CTC treatment (Fig. S10), although the acid contents did not differ significantly (Fig. S4).

AA, an unsupervised ML method, was used to identify items related to the presence or absence of CTC (Fig. S11). Since AA excels at using the same criteria for evaluating across hierarchies, all bacterial taxa, physiological data, and daily ages were used as computational data. AA showed increases in growth performance indicators, including the body weight (BW), heart girth, waist circumference, and plasma IGF-1 concentration. In addition, increased abundance of the genera *Methanosphaera* and *Methanobrevibacter*, which belong to the family *Methanobacteriaceae*, were associated with treatment with CTC, consistent with the LDA results.

**Figure 4:**
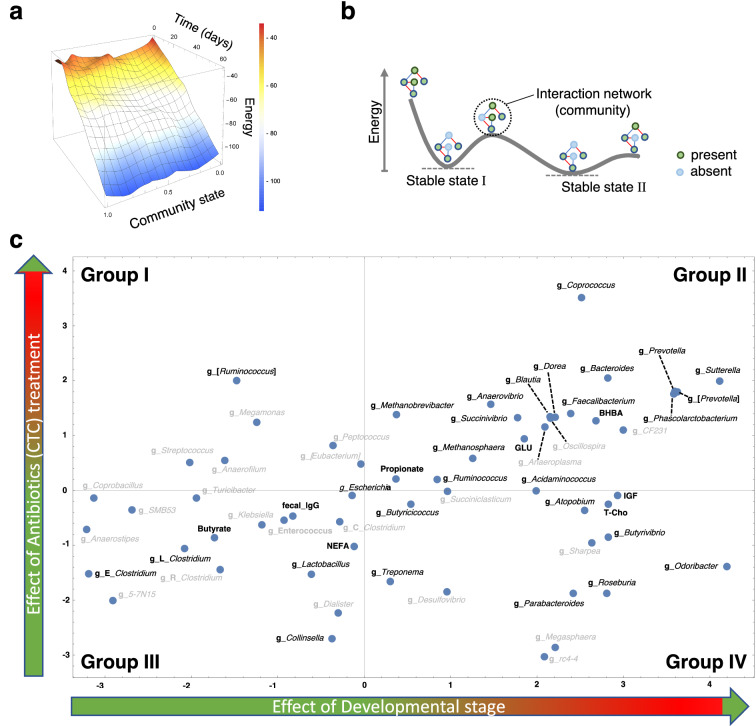
Screening for potential relationships among factors for energy landscape analysis (ELA) and refinement by other machine learning (ML) approaches. (**a**) The ELAassociated with antibiotic treatment is visualized. The axis formed the energy landscape with compositional energy, community state, and treatment time (days). (**b**) The concept of the stable state in ELA is shown. Each green circle indicates a constituent element (component) within an interaction network (community). The blue and red lines show positive and negative effects between the components, respectively. Each interaction network was different depending on the energy state. (**c**) Response to environmental ε. Dependencies on the developmental stage (${g}_{i}^{d}$) (X axis) and responsiveness to antibiotic treatment (${g}_{i}^{a}$) (Y axis) are plotted. Four categories are shown as Groups I-IV. The bacteria categorized within Group I had low population levels at 30–60 d that increased after with antibiotic treatment. Those within Group II had high population levels at 30–60 d that increased with antibiotic treatment. Those within Group III had low population levels at 30–60 d that decreased with antibiotic treatment. Those within Group IV had high population levels at 30–60 d and/or an independent population at this stage, but the population levels decreased with antibiotic treatment. The black letters indicate the bacterial genera and the other physiological components selected by LDA and AA.

The overall characteristics of the bacterial flora and physiological indicators of the CTC-treated and untreated individuals were evaluated by topography based on ELA as an unsupervised ML method. ELA showed the stability of the physiological components throughout the duration of the experiment (Fig. 4a) due to the absence of a typical stable state (Fig. 4b), as previously described^13^. These characteristics appeared to be related to the seemingly small number of physiological changes, such as expressivity, as described above (Figs. 2a and S1–S4). However, based on the correlation analysis and the LDA and AA results, the existence of potential changes that were not detected by general statistical analysis can be inferred. Therefore, the relationships among factors related to the effects of CTC on growth were evaluated. The effects of antibiotic administration (Y-axis) and growth (X-axis) classified unstable groups (four groups) after exposure to CTC (Fig. 4c). It appeared difficult to find any regularity between the groups of components selected for LDA and AA that would indicate a relationship between them. Therefore, to reevaluate the potential impacts, the analysis was performed using a method that utilizes statistical causal inference, as follows.

### Assessment by statistical causal inference

Statistical causal inference based on LiNGAM analysis for each group inferred that the relationship of bacteria was different between the groups with and without CTC administration (Figs. S12 and S13).

Statistical causal inference was performed on the group of factors characteristically in the category with CTC treatment sorted by AA (Fig. S11a). As described in Fig. S12a, the calculation with the CON data inferred that the presence of CTC had a significantly negative effect (−2104.03) on butyrate production. The genus *Escherichia* was inferred to have a significant positive effect (4320.78) on butyrate production. It was inferred that these values were correct estimations with high probability because the relationship with the higher taxa family *Enterobacteriaceae* for the genus *Escherichia* regarded as a potential internal standard was 1. The results suggested that several groups of bacteria had an effect on these relationships. Concomitantly, antibiotic administration also had a negative effect on the serum IgA levels (−221.17). It was inferred that the genus *Clostridium* belonging to the family *Lachnospiraceae* had an extremely positive effect (+ 283,812.13) on an increase in the serum IgA levels. The genera *Faecalibacterium* and *Succinivibrio* also had positive effects (+ 3231.67 and + 12,835.36). The genus *Succinivibrio* was estimated to have a strong positive effect (+ 42,234.56) on propionate production. In contrast, statistical causal inference on the same group of factors (Fig. S11a) with the EXP data found a different causal relationship between the two groups. (Fig. S12b). In other words, the group of factors characterized by CTC treatment was not involved in the production of butyrate, cholesterol, and serum IgA, at least in the EXP group.

Subsequently, statistical causal inference was performed on the group of factors characteristically in the category without antibiotic treatment sorted by AA (Fig. S11b). An analysis of the data of only the CON group inferred that the family *Lachnospiraceae*, selected by LDA, and the genus *Lactobacillus* might have a weak negative effect (−1.20 and −1.93, respectively) on the effect of CTC (Fig. S13a). Since the values of the family *Prevotellaceae* and the genus *Prevotella*, which are positioned as internal standards, were 0.99, this calculation was inferred to be the correct titer. When only the data of the EXP were analyzed, the genus *Clostridium* belonging to *Erysipelotrichaceae* and the genus *Lactobacillus* were estimated to have positive effects on the family *Lachnospiraceae* (+ 5.80 and + 0.74, respectively). The internal standard at this time was 0.95 for the values between the family *Prevotellaceae* and the genus *Prevotella*.

**Figure 5:**
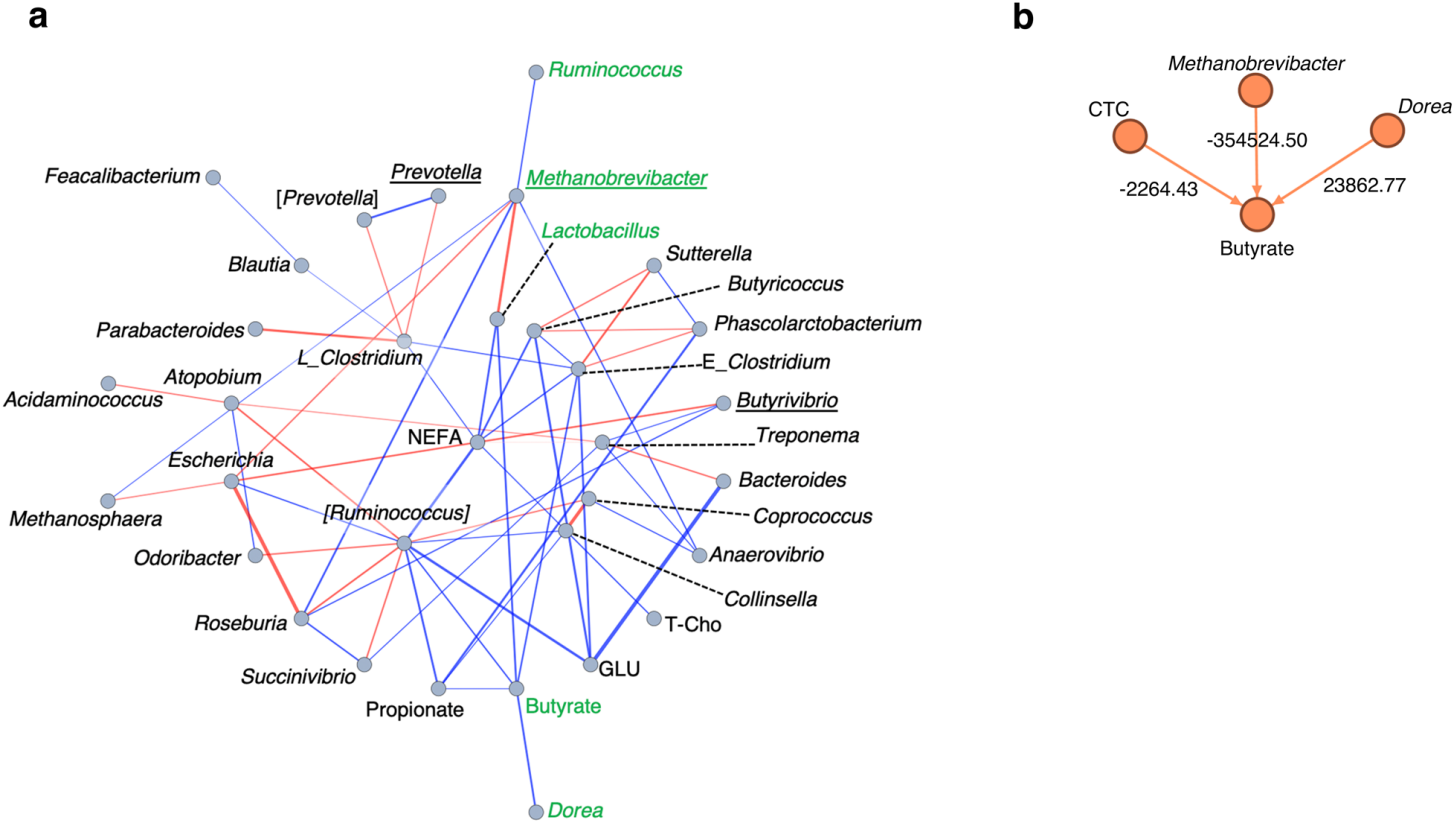
(**a**) The ELA interaction network shows the significant relationships in the extended pairwise maximum entropy model fitted to the observational data. Components were selected by linear discriminant analysis and association analysis. The bacteria selected by both analyses are underlined. The blue and red lines show positive and negative relationships, respectively. The abbreviations are as follows: E: family *Erysipelotrichacea*e; L: family *Lachnospiraceae*; GLU: serum glucose; NEFA: serum nonesterified free fatty acid; butyrate: fecal butyric acid; propionate: fecal propionic acid; T-Cho: serum total cholesterol. (**b**) The calculated causal relationship of the components strongly linked with butyrate is visualized by linear non-Gaussian acyclic model (DirectLiNGAM) analysis. The amounts of change (d 3, 30, and 60) with respect to the values at d 3 of the components aligned with butyrate (green letters) (**a**) were used for the calculation. The arrow shows the trend of the causal relationship. The number shows the value of the causal contribution calculated by the DirectLiNGAM analysis. The minus and plus values show negative and positive causal contributions, respectively.

Furthermore, a computational evaluation of the interactions among the components selected by three types of ML methods was conducted at the genus level. ELA interaction network analyses visualized the relationship among the ML-selected components as statistical physics (Fig. 5a). These data from statistical network analysis showed complex positive and negative relationships among complex bacterial groups and physiological factors. In particular, the network estimated an adverse effect of the genus *Methanobrevibacter* on the genus *Lactobacillus* abundance and fecal butyrate concentration.

Based on these results, causal inference by DirectLiNGAM was conducted considering growth stages, focusing on a network affecting butyrate production. On the ELA network line of the genus *Methanobrevibacter* on the genus *Lactobacillus* and the fecal butyrate concentration, the causal inference estimated the negative causality for butyrate production by CTC treatment and the genus *Methanobrevibacter* (Fig. 5b), together with the positive causality for the genus *Dorea* belonging to the family *Lachnospiraceae*. These numerical contributions of the directed acyclic graphs (DAGs) were large for butyrate production, suggesting that they are important as potential negative effects of CTC as antibiotics.

Thus, the results of the ELA and their causal inference suggested that complex networks of antibiotic treatment and the gut microbiota may have potential control over physiological effects such as butyrate production.

## Discussion

First, the present study aimed to show that feeding Japanese Black calves without antibiotic supplementation does not adversely affect performance, as indicated by the fact that general statistical analyses did not detect significant differences in the phenotypes (growth rate, feed efficiency, and the levels of serum and fecal components) between calves with/without CTC administration. However, CTC treatment altered the weighted UniFrac distances at 60 d and the correlations among the major fecal bacteria, organic acid levels, and phosphate levels. In addition, LDA revealed that CTC treatment affected the populations of various types of fecal bacteria. Furthermore, AA revealed that CTC administration resulted in increased growth performance parameters, which was not contradictory with the results of previous studies showing the positive impact of antibiotics on growth in ruminants^4^. These findings indicate that CTC administration potentially improves calf growth performance via alterations in the intestinal environment. Similarly, a previous study found that the administration of antibiotics at low concentrations affected populations of the gut microbiota in preweaning calves^14^.

Organic acids, including SCFAs, are the primary end products of the intestinal microbiota and are subsequently utilized by the host as substrates for the production of metabolic energy^15^. Therefore, alterations in the microbiota populations are associated with SCFA production in the intestine, which can affect host productivity and health. In this study, the causal inference based on ML estimated that the fecal butyrate and serum IgA concentrations were negatively affected by CTC administration (Figs. S12a and S13a). Furthermore, these estimations inferred that high abundance of the genus *Lactobacillus* and/or family *Lachnospiraceae*, a butyrate-producing bacteria^16^, was negatively associated with the antibiotic treatment (Figs. S12a and S13a). This trend was also confirmed by the ELA focused on the genus level (Fig. 5a): positive causality was observed between fecal butyrate levels and the abundance of genus *Dorea* belonging to the family *Lachnospiraceae* (Fig. 5b). Thus, CTC administration seemed to decrease the abundance of butyrate-producing bacteria, resulting in a decreased butyrate concentration in feces. Similar to the present findings, Shang et al.^17^ reported that piglets with a greater fecal abundance of the family *Lachnospiraceae* and fecal butyrate concentration had higher fecal IgA, IgG, and IgM concentrations. Butyrate has been shown to exert various beneficial effects, including enhanced intestinal development, improved barrier function, mitigation of inflammatory responses, and alteration of the intestinal microbiota^18,19^. Therefore, it is possible that a high abundance of the family *Lachnospiraceae* is associated with an improved immune response through fecal butyrate. Therefore, it was inferred that the effect was more pronounced in the absence of antibiotics than in the presence of antibiotics.

In particular, AA estimated that the abundance of the genus *Methanobrevibacter*, a major group of methanogenic bacteria accounting for approximately two-thirds of the archaea in the rumen^20^, was associated with CTC administration, consistent with the LDA results. An increased population of methanogens is involved in the development of obesity in humans^15^. In humans, methanogenic archaea have been reported to be highly resistant to antibiotics^21^, which may explain the increased methanogens in calves administered CTC. Enteric methane emissions are a problem due to energy loss, adverse effects on animal productivity, and environmental issues^15,22^. Furthermore, the methane emissions from cattle cause a loss of enteric methane energy, accounting for 2–12% of the gross energy intake^23^. Thus, an increased abundance of methanogens due to CTC administration has the potential to adversely affect cattle productivity. Interestingly, DirectLiNGAM analysis inferred that the fecal butyrate concentration exhibited a marked negative relationship with the abundance of the genus *Methanobrevibacter*. These observations provide a research perspective on improving a combination of aspects, including mucosal immunity and environmental impact, as well as lipid accumulation as a growth quality.

We previously reported that *Caldibacillus hisashii*, a member of the class *Bacilli*, was associated with a marked reduction in the abundance of the genus *Methanobrevibacter*^15^*.* In the present study, the class *Bacilli* attenuated the effects of antibiotics, as estimated by LiNGAM analysis (Fig. S14a). Furthermore, LiNGAM analysis estimated a positive causality for the abundance of the family *Lachnospiraceae* and the fecal butyrate concentration (Fig. S14b) with the abundance of the class *Bacilli*. Therefore, it is possible that the negative effect of antibiotic administration on the concentration of fecal butyrate was due in part to an increase in the abundance of methanogens. A previous study^24^ found a negative correlation between the mean concentration of fecal butyrate and the abundance of methanogens, similar to the result in the present study. Previous studies, which investigated the syntrophic relationship between butyrate-degrading bacteria and methanogens, have revealed that methanogen inhibitors prevent butyrate degradation in thermophilic mixed cultures^25,26^. Methanogen-assimilated hydrogen is utilized in methanogenesis but not in the colon^24^. In contrast, hydrogen assimilated by hydrogenotrophic acetogens is utilized to convert CO_2_ to acetate and may contribute to the fecal butyrate concentration through bacteria that produce butyrate^24^. Therefore, the negative relationship between methanogens and butyrate is likely to involve competition for colonic hydrogen between methanogens and hydrogenotrophic acetogens. The verification of these previous studies, including the current study, will be an issue to address in future research.

Overall, this study successfully clarified the potential impact of antibiotic administration by combining complex machine learning and causal inference to assess physiological dynamics that are difficult to understand by a general comparative analysis. These observations imply an instability in homeostatic plasticity with respect to calf growth and the potential environmental load by antibiotics. The current study first highlighted the possibility that antibiotic administration to promote the growth rate decreased intestinal butyrate production through alterations in the population of butyrate-producing bacteria and methanogens. The estimates from this study may have significant implications for improving livestock productivity and reducing the environmental load of antibiotics and global warming gases. More research is needed to unravel these concerns.

## Methods

### Animal management

Twelve Japanese Black calves (8 male and 4 female calves) were managed with the respective dams until 3 days (d) of age. Subsequently, the calves were separated from the dams and moved to calf pens at 4 d of age. Milk replacer (Calf Top EX Black, Zen-Raku-Ren, Tokyo, Japan) was offered to the calves starting from 4 d of age using an automated calf feeder (Forster Technique, Co., Ltd., Germany). The calves were randomly assigned to one of two treatment groups [CON group: n = 6 (4 male and 2 female calves), initial body weight (BW) = 31.5 ± 5.1 kg; EXP group: n = 6 (4 male and 2 female calves), initial BW = 30.8 ± 5.1 kg]: the calves in the CON group were fed with milk replacer containing CTC at 10 g/kg, whereas those in the EXP group were fed with antibiotic-free milk replacer. All milk replacers were formulated to contain 28.0% crude protein (CP), 18.0% crude fat (CF), and 108.0% total digestible nutrients (TDNs). The amount of milk replacer offered was increased from 0.5 kg/d to 1.0 kg/d from 4 to 21 d of age and maintained at this level until 60 d of age. All calves were fed the same calf starter containing 18.0% CP, 2.0% CF, and 72.5% TDNs (Hello Starter, Zen-Raku-Ren, Tokyo, Japan) and hay containing 12.4% CP, 4.1% CF, and 62.0% TDNs ad libitum starting from 4 d of age. No diets, except for the CON milk replacer, contained antibiotics. Throughout the study, all the calves had free access to water and mineral blocks (Cowstone A, Nippon Zenyaku kogyo Co. Ltd., Fukushima, Japan). The individual feed intake was recorded daily, and the BW was measured every month. The present study was conducted at a Kuju agricultural research center (Oita, Japan), where Japanese Black cattle are raised. All experimental protocols were approved by the Kyushu University Laboratory Animal Care and Use Committee (approval no. A30-355-1). The procedures used in the present study were performed according to the ARRIVE guidelines and the Guidelines for Animal Experiments by the Faculty of Agriculture at Kyushu University.

### Sample collection

On the day before sampling (2, 29, and 59 d of age), an automated calf feeder was not available for the calves. Blood samples were collected at 3, 30, and 60 d of age immediately before the morning feeding at 9 a.m.; this was achieved using Vacutainers to collect serum (Venoject® II, Terumo Co., Ltd., Tokyo, Japan). All the samples were centrifuged at 1200 × g for 30 min at 20 °C, and serum was stored at −80 °C until analysis. Single-use gloves disinfected with 70% ethanol and sampling tubes with spoons were used to collect rectal feces from the calves immediately after blood sampling at 3, 30, and 60 d of age. The fecal samples were stored at − 30 °C and thereafter stored at − 60 °C to − 80 °C until analysis.

### Analyses of serum hormones and metabolites

The serum concentrations of glucose, total cholesterol (T-Cho), nonesterified fatty acids (NEFAs), β-hydroxybutyric acid (BHBA), urea nitrogen (SUN), and Ca^2+^ were measured using commercially available assay kits by the glucose oxidase enzymatic method (glucose B-test; Wako Pure Chemical, Osaka, Japan), the acyl-CoA synthetase-acyl-CoA oxidase enzymatic method (FFAC; Wako Pure Chemical), the ACS-ACOD method (NEFA C-test, Wako Pure Chemical Industries, Ltd., Japan), a bovine beta-hydroxybutyric acid (β-OHB) ELISA kit (Huamei Biotech Co., Ltd., Wuhan, China), the enzymic method (DetectX Urea Nitrogen Colorimetric Detection Kit, Arbor Assays LLC, USA), and the OCPC method (Metallo Assays Calcium, Metallogenics Co., Ltd., Japan), respectively, according to the manufacturer’s instructions. The serum concentration of insulin-like growth factor 1 (IGF-1) was measured by time-resolved fluoroimmunoassay as previously described^27,28^.

### HPLC analysis of fecal metabolic acids

Fecal samples (200–400 mg) were prepared according to a previously described protocol^29^ with some modifications. Briefly, the samples were mixed with a ninefold volume of Milli-Q water for 10 min. After centrifugation at 15,000 rpm, all of the supernatants were filtered with 0.45-μm filters (Millex-HA Filter Unit SLHA025NB; Merck). The filtered solutions were subjected to high-performance liquid chromatography (HPLC) analysis. To determine the concentrations of lactic acid, acetic acid, propionic acid, butyric acid, valeric acid, isovaleric acid, and phosphoric acid, frozen fresh fecal samples were analyzed using an HPLC Prominence instrument (Organic Acid Analyzer; Shimadzu, Kyoto, Japan) was equipped on an ion-exclusion column (Shim-pack SCR-102H; Shimadzu) and an electric conductivity detector (CDD-10AVP; Shimadzu). The analytical conditions were as follows: mobile phase, 5 mM *p*-toluenesulfonic acid; buffer, 5 mM *p*-toluenesulfonic acid, 20 mM Bis–Tris, and 0.2 mM EDTA-4H; temperature, 40 °C; and flow rate, 0.8 ml/min.

### Meta-sequence analysis of bacterial 16S rRNA gene sequences

The fecal samples from the CON (with antibiotic treatment) and EXP (without antibiotic treatment) groups were used for DNA extraction using a QIAamp PowerFecal DNA Kit (QIAGEN N.V., Inc.) according to the manufacturer’s protocol. The DNA concentration was evaluated using a Quant-iT™ PicoGreen dsDNA Assay Kit (Thermo Fisher Scientific). The V4 region of the bacterial 16S rRNA gene (515F-806R) was sequenced according to a previous study^15,30^. The obtained sequences were filtered by Trimmomatic (http://www.usadellab.org/cms/?page=trimmomatic), and 5000 trimmed reads per sample were analyzed with QIIME 1.9.1. The α-diversity, β-diversity, bacterial community, and correlations were visualized using the packages “genefilter”, “gplots”, “ggplot2”, “RColorBrewer”, “pheatmap”, “ape”, “base”, “dplyr”, “easyGgplot2”, “knitr”, “ggthemes”, “phyloseq”, and “vegan” in R software (versions 4.0.5). The number of observed OTUs and the Chao1, Shannon, and Simpson index values were assessed as measures of α-diversity.

The β diversities were estimated by principal coordinate analysis (PCoA) using weighted or unweighted UniFrac distances based on the OTU distribution across samples. These statistical values of β diversities were analyzed using the function ‘adonis’ of the R software packages "vegan". All 16S rRNA gene datasets were deposited in the GenBank Sequencing Read Archive database (accession number: DRA010973; BioSample numbers: SAMD00252058-SAMD00252093/SSUB016265).

### LDA

LDA is an elementary method of supervised ML. Here, LEfSe was used to identify genomic taxa characterizing the differences between the experimental conditions. LDA score plots and the cladogram based on LEfSe were visualized by Galaxy (https://huttenhower.sph.harvard.edu/galaxy/) as described in a previous overview^31^. The populations of predominant bacterial community members (more than 0.1% of the total bacterial population) were analyzed. The threshold of the logarithmic LDA score for discriminative features was set to 3.0. The value provided an LDA-cladogram and LDA-score plots for the factorial Kruskal‒Wallis test among classes and the value for the pairwise Wilcoxon test between subclasses (set at 0.05) as nonparametric analyses. The pairwise comparisons among subclasses to be performed only among subclasses with the same name were set to “Yes”. The strategy for multiclass analysis was set to “All-against-all (more strict)”.

### ELA

ELA, an elementary method of unsupervised ML, was performed as previously described^13,32^. ELA is a data-driven method for constructing landscapes that explain the stability of community compositions across environmental gradients. Here, ELA was based on an extended pairwise maximum entropy model that explains the probability of the occurrence of the ecological state of sample $k$, ${\sigma }^{\left(k\right)}$ given the environmental condition ${\epsilon }^{(k)}$; as the ecological state, we combined the presence/absence status of selected taxa and levels of physiological factors, ${\sigma }^{\left(k\right)}=\left({\sigma }_{1}^{\left(k\right)},{\sigma }_{2}^{\left(k\right)},\dots {\sigma }_{N}^{\left(k\right)}\right)=\left({\sigma }_{1}^{\left(k\right)},\dots ,{\sigma }_{{n}_{m}}^{\left(k\right)},{\sigma }_{N-{n}_{c}}^{\left(k\right)}\dots {\sigma }_{N}^{\left(k\right)}\right)$ where ${n}_{m}$ is the number of bacterial taxa and ${n}_{c}$ is the number of chemicals; as the environmental condition, two environmental factors represented as with (1) or without (0) antibiotic treatment (${\epsilon }_{a,i}^{(k)}$) and growth stages converted to the 0–1 range (${\epsilon }_{s,i}^{(k)}$; 3, 30 and 60 d were converted to 0, 0.53 and 1, respectively) were combined as ${\epsilon }^{(k)}=({\epsilon }_{a,i}^{(k)},{\epsilon }_{s,i}^{(k)})$. The model can be written as follows:

1 $$P\left({\sigma }^{\left(k\right)}|{\epsilon }^{\left(k\right)}\right)=\frac{{e}^{-E\left({\sigma }^{\left(k\right)}|{\epsilon }^{\left(k\right)}\right)}}{{\Sigma e}^{-E\left({\sigma }^{\left(k\right)}|{\epsilon }^{\left(k\right)}\right)}},$$

2 $$E\left({\sigma }^{\left(k\right)}|{\epsilon }^{(k)}\right)=-\left({\Sigma }_{i}{\Sigma }_{j}{J}_{ij}{\sigma }_{i}^{\left(k\right)}{\sigma }_{j}^{\left(k\right)}+{\Sigma }_{i}{g}_{i}^{a}{\epsilon }_{a,i}^{\left(k\right)}{\sigma }_{i}^{\left(k\right)}+{\Sigma }_{i}{g}_{i}^{s}{\epsilon }_{s,i}^{\left(k\right)}{\sigma }_{i}^{\left(k\right)}+{\Sigma }_{i}{h}_{i}{\sigma }_{i}^{\left(k\right)}\right).$$

Here, $P({\sigma }^{\left(k\right)}|\epsilon )$ is the probability of the occurrence of an ecological state ${\sigma }^{\left(k\right)}$. Equation (1) shows that the probability is high when energy $E\left({\sigma }^{\left(k\right)}|\epsilon \right)$ is low and vice versa. In Eq. (2), $E\left({\sigma }^{\left(k\right)}|\epsilon \right)$ is defined as the sum of the effect of interaction among components, antibiotic treatment, growth stages, and the net effect of unobserved environmental factors. The parameters in eq. (II), namely, ${J}_{ij}$, ${g}_{i}^{a}$, ${g}_{i}^{s}$, and ${h}_{i}$, indicate the effect of the relationship among components (${J}_{ij}>0$ favors and ${J}_{ij}<0$ disfavors the cooccurrence of components $i$ and $j$), the effect of the antibiotics on component $i$ (the antibiotic treatment positively (${g}_{i}^{a}>0$) or negatively (${g}_{i}^{a}<0$) affects the occurrence of component $i$), the effect of the growth stages on component $i$ (component $i$ favors the later (${g}_{i}^{s}>0$) or early (${g}_{i}^{s}<0$) growth stage) and how likely component $i$ occurs when the other factors are equal, respectively.

All the components in ${\sigma }^{\left(k\right)}$ were converted to the 0–1 range as follows. We first interpreted the relative abundance of each bacterial genus in the samples as presence (1) or absence (0) states by setting a threshold value of 0.001. In this study, we selected 36 genera that appeared in more than 2 samples but fewer than 35 samples. We combined these into the density of eight selected chemicals, which were converted to the 0–1 range. Accordingly, for each of the 36 samples, we obtained the set of explanatory variables ${\sigma }^{\left(k\right)}$ with 44 components, which accompanied the environmental condition ${\epsilon }^{(k)}$ representing the status of antibiotic treatment and growth stage.

The parameters *h*_*i*_, ${J}_{ij}$, ${g}_{i}^{a}$ and ${g}_{i}^{s}$ were estimated based on a stochastic approximation algorithm^13,33,34^. The stochastic approximation estimates the expected values of the sufficient statistics by averaging over a more manageable number of simulated assemblages during each model-fitting iteration while still retaining maximum likelihood convergence. These detailed procedures are described in the Supplementary Methods (“ELA interaction network” as a subheading). In brief, the algorithm was repeated 2000 times to obtain the p values for each parameter. Then, $J$ s were used to reconstruct an ELA interaction network.

### DirectLiNGAM

To estimate a structural model beyond the distribution of limited experimental data^30^, A direct method for learning a linear non-Gaussian acyclic model (DirectLiNGAM) approach^32,35,36^ involves independent component analysis and a non-Gaussian method for estimating causal structures. In this study, the properties of DirectLiNGAM used in independent components were exploited. Specifically, the data of the bacterial population were not separated from the data of the same hierarchy (class, or family and genus) detected by ML; thus, the calculation results could be evaluated as an internal standard. The fact that the calculation results were close to 1 for the data of the same hierarchy was verified. DirectLiNGAM (https://github.com/cdt15/lingam) was established with Python (version 3.7.10) on CentOS (version 7.9).The data calculated by DirectLiNGAM analysis were confirmed as DAGs by the python library “numpy (version 1.21.5)”, “pandas (version 1.4.3)”, “matplotlib (version 3.5.2)”, “lingam (version 1.5.4)”, and “graphviz (version 0.19.1)”. Based on the data of DAGs, arranged DAGs were visualized by Gephi (version 0.9.2) (https://gephi.org).

### Statistical analyses

In addition to LDA, AA, ELA, and LiNGAM analysis, individual data were analyzed using the MIXED procedure of JMP14 (SAS Institute Inc. Cary, NC, USA) according to the following model:

$${\text{Y}}_{{{\text{ijk}}}} =\upmu + {\text{G}}_{{\text{i}}} + {\text{T}}_{{\text{j}}} + {\text{C}}_{{\text{k}}} + {\text{GT}}_{{{\text{ij}}}} + {\text{e}}_{{{\text{ijk}}}}$$

where Y_ijk_ is the dependent variable, μ is the overall mean, G_i_ is the fixed effect of treatment, T_j_ is the fixed effect of time (age) used as a repeated measure, GT_ij_ is the fixed effect of the interaction of treatment with time after birth, C_k_ is the random effect of the calf, and e_ijk_ is the error term. A simple main effect test was performed to detect the differences between groups at the same point. Significance was declared if *P* < 0.05, and a tendency was assumed if 0.05 ≤ *P* < 0. 20. The relative values of dominant and/or characteristic bacteria were visualized through construction of a correlation graph and heatmap after Pearson’s correlation coefficient was calculated for the selected bacteria (> 1% of the detected community and > 0.1% of the community selected by LDA) using R software (version 4.0.5). The data are presented as the means ± SDs.

### Other methods

Information for the analysis of the serum and fecal IgA, IgG, and IFN-γ concentrations, AA, and the analyses based on the data shown in Data S1 are described in the Supplementary Methods.

## Supplementary Information

Supplementary Information 1.

Supplementary Information 2.

Supplementary Information 3.

## Data Availability

The datasets presented in this study can be found as Excel files (named DataS1 FINAL.xlsx) in the Supplementary Information. Furthermore, all 16S rRNA gene datasets have been deposited in the DDBJ Sequence Read Archive and can be found under accession number DRA010973. In addition, the R protocols for AA used in this study were deposited on the following websites (named Market Basket Analysis): http://dmar.riken.jp/Rscripts/ and http://dmar.riken.jp/NMRinformatics/.

## References

[1] Moore PR, Evenson A (1946). Use of sulfasuxidine, streptothricin, and streptomycin in nutritional studies with the chick. J. Biol. Chem..

[2] Cromwell GL (2002). Why and how antibiotics are used in swine production. Anim. Biotechnol..

[3] Barlow GM, Yu A, Mathur R (2015). Role of the gut microbiome in obesity and diabetes mellitus. Nutr. Clin. Pract..

[4] Bartley EE, Fountaine FC, Atkeson FW, Fryer HC (1953). Antibiotics in dairy cattle nutrition. I. The effect of an aureomycin product (Aurofac) on the growth and well-being of young dairy calves. J. Dairy Sci..

[5] Woolhouse ME, Ward MJ (2013). Microbiology. Sources of antimicrobial resistance. Science.

[6] Van Boeckel TP (2017). Reducing antimicrobial use in food animals. Science.

[7] Mazmanian SK, Liu CH, Tzianabos AO, Kasper DL (2005). An immunomodulatory molecule of symbiotic bacteria directs maturation of the host immune system. Cell.

[8] Peterson DA, McNulty NP, Guruge JL, Gordon JI (2007). IgA response to symbiotic bacteria as a mediator of gut homeostasis. Cell Host Microbe.

[9] Claesson MJ (2011). Composition, variability, and temporal stability of the intestinal microbiota of the elderly. Proc. Natl. Acad. Sci. USA.

[10] Looft T (2012). In-feed antibiotic effects on the swine intestinal microbiome. Proc. Natl. Acad. Sci. USA.

[11] Robinson K (2019). Differential impact of subtherapeutic antibiotics and ionophores on intestinal microbiota of broilers. Microorganisms.

[12] Brown K, Zaytsoff SJ, Uwiera RR, Inglis GD (2016). Antimicrobial growth promoters modulate host responses in mice with a defined intestinal microbiota. Sci. Rep..

[13] Suzuki K, Nakaoka S, Fukuda S, Masuya H (2021). Energy landscape analysis elucidates the multistability of ecological communities. Ecol. Monogr..

[14] Yousif MH (2018). Low concentration of antibiotics modulates gut microbiota at different levels in pre-weaning dairy calves. Microorganisms.

[15] Inabu Y (2022). Development of a novel feeding method for Japanese black calves with thermophile probiotics at postweaning. J. Appl. Microbiol..

[16] Zhang J (2019). Beneficial effect of butyrate-producing Lachnospiraceae on stress-induced visceral hypersensitivity in rats. J. Gastroenterol. Hepatol..

[17] Shang Q, Liu H, Wu D, Mahfuz S, Piao X (2021). Source of fiber influences growth, immune responses, gut barrier function and microbiota in weaned piglets fed antibiotic-free diets. Anim. Nutr..

[18] Bedford A, Gong J (2018). Implications of butyrate and its derivatives for gut health and animal production. Anim. Nutr..

[19] Furusawa Y (2013). Commensal microbe-derived butyrate induces the differentiation of colonic regulatory T cells. Nature.

[20] Janssen PH, Kirs M (2008). Structure of the archaeal community of the rumen. Appl. Environ. Microbiol..

[21] Dridi B, Fardeau ML, Ollivier B, Raoult D, Drancourt M (2011). The antimicrobial resistance pattern of cultured human methanogens reflects the unique phylogenetic position of archaea. J. Antimicrob. Chemother..

[22] Subepang S, Suzuki T, Phonbumrung T, Sommart K (2019). Enteric methane emissions, energy partitioning, and energetic efficiency of zebu beef cattle fed total mixed ration silage. Asian-Australas J. Anim. Sci..

[23] Gerber PJ (2013). Technical options for the mitigation of direct methane and nitrous oxide emissions from livestock: A review. Animal.

[24] Abell GCJ, Conlon MA, McOrist AL (2006). Methanogenic archaea in adult human faecal samples are inversely related to butyrate concentration. Microb. Ecol. Health Dis..

[25] Ahring BK, Westermann P (1987). Thermophilic anaerobic degradation of butyrate by a butyrate-utilizing bacterium in coculture and triculture with methanogenic bacteria. Appl. Environ. Microbiol..

[26] Zhang C, Liu X, Dong X (2005). Syntrophomonas erecta sp. Nov., a novel anaerobe that syntrophically degrades short-chain fatty acids. Int. J. Syst. Evol. Microbiol..

[27] Sugino T (2004). Effects of ghrelin on food intake and neuroendocrine function in sheep. Anim. Reprod. Sci..

[28] Laarman AH, Ruiz-Sanchez AL, Sugino T, Guan LL, Oba M (2012). Effects of feeding a calf starter on molecular adaptations in the ruminal epithelium and liver of Holstein dairy calves. J. Dairy Sci..

[29] Poudel P (2017). Development of a systematic feedback isolation approach for targeted strains from mixed culture systems. J. Biosci. Bioeng..

[30] Miyamoto H (2022). A potential network structure of symbiotic bacteria involved in carbon and nitrogen metabolism of wood-utilizing insect larvae. Sci. Total Environ..

[31] Segata N (2011). Metagenomic biomarker discovery and explanation. Genome Biol..

[32] Miyamoto H (2023). Computational estimation of sediment symbiotic bacterial structures of seagrasses overgrowing downstream of onshore aquaculture. Environ. Res..

[33] Harris DJ (2016). Inferring species interactions from co-occurrence data with Markov networks. Ecology.

[34] Salakhutdinov R, Hinton G (2012). An efficient learning procedure for deep Boltzmann machines. Neural Comput..

[35] Shimizu, S., Hoyer, P. O., Hyvarinen, A. & Kerminen, A. A Linear Non-Gaussian Acyclic Model for Causal Discovery. *J. Mach. Learn. Res.*, 2003–2030 (2006).

[36] Shimizu S (2011). DirectLiNGAM: A direct method for learning a linear non-Gaussian structural equation model. J. Mach. Learn. Res..

