# Supplementary material for: Estimation of silent phenotypes of calf antibiotic dysbiosis
Source: Sci Rep. 2023 Apr 19;13:6359. doi: 10.1038/s41598-023-33444-0 (PMC10115819; doi:10.1038/s41598-023-33444-0)
Supplement: Supplementary file 1 — Supplementary Information 1. [file 41598_2023_33444_MOESM1_ESM.pdf]

Supplementary Information

Estimation of silent phenotypes of calf antibiotic dysbiosis

Shunnosuke Okada[1], Yudai Inabu[1], Hirokuni Miyamoto[2,3,4,5], Kenta Suzuki[6], Tamotsu Kato[3], Atsushi Kurotani[7,8], Yutaka Taguchi[1], Ryoichi Fujino[1], Yuji Shiotsuka[1], Tetsuji Etoh[1], Naoko Tsuji[5], Makiko Matsuura[2,5], Arisa Tsuboi[4,5,7], Akira Saito[9], Hiroshi Masuya[6], Jun Kikuchi[7], Yuya Nagasawa[10], Aya Hirose[10], Tomohito Hayashi[10], Hiroshi Ohno[3]*, Hideyuki Takahashi[1]*.*

[1]*Kuju Agricultural Research Center, Graduate School of Agriculture, Kyushu University, Oita, Japan, 878-0201*
[2]*Graduate School of Horticulture, Chiba University, Chiba, Japan, 263-8522*
[3]*RIKEN Integrated Medical Science Center, Yokohama, Kanagawa, Japan, 230-0045*
[4]*Japan Eco-science (Nikkan Kagaku) Co., Ltd., Chiba, Japan, 260-0034*
[5]*Sermas, Co., Ltd., Chiba, Japan, 271-8501*
[6]*RIKEN, BioResource Research Center, Tsukuba, Ibaraki, Japan, 305-0074*
[7]*RIKEN Center for Sustainable Resource Science, Yokohama, Kanagawa, Japan, 230-0045*
[8]Research Center for Agricultural Information Technology, National Agriculture and Food Research Organization, Tsukuba, Ibaraki, Japan, 305-0856
[9]*Feed-Livestock and Guidance Department, Dairy Technology Research Institute, The National Federation of Dairy Co-operative Associations (ZEN-RAKU-REN), Fukushima, Japan*
[10]*Pathology and Production Disease Group, Division of Hygiene Management, Hokkaido Research Station, National Institute of Animal Health, National Agriculture and Food Research Organization, Hokkaido, Japan, 062-0045.*

* Cocorrespondence:
Hirokuni Miyamoto Ph.D., hirokuni.miyamoto@riken.jp, h-miyamoto@faculty.chiba-u.jp
Hiroshi Ohno Ph.D. and M.D., RIKEN IMS, hiroshi.ohno @riken.jp
Hideyuki Takahashi Ph.D., takahashi.hideyuki.990@m.kyushu-u.ac.jp

This file includes: Figures S1 to S14

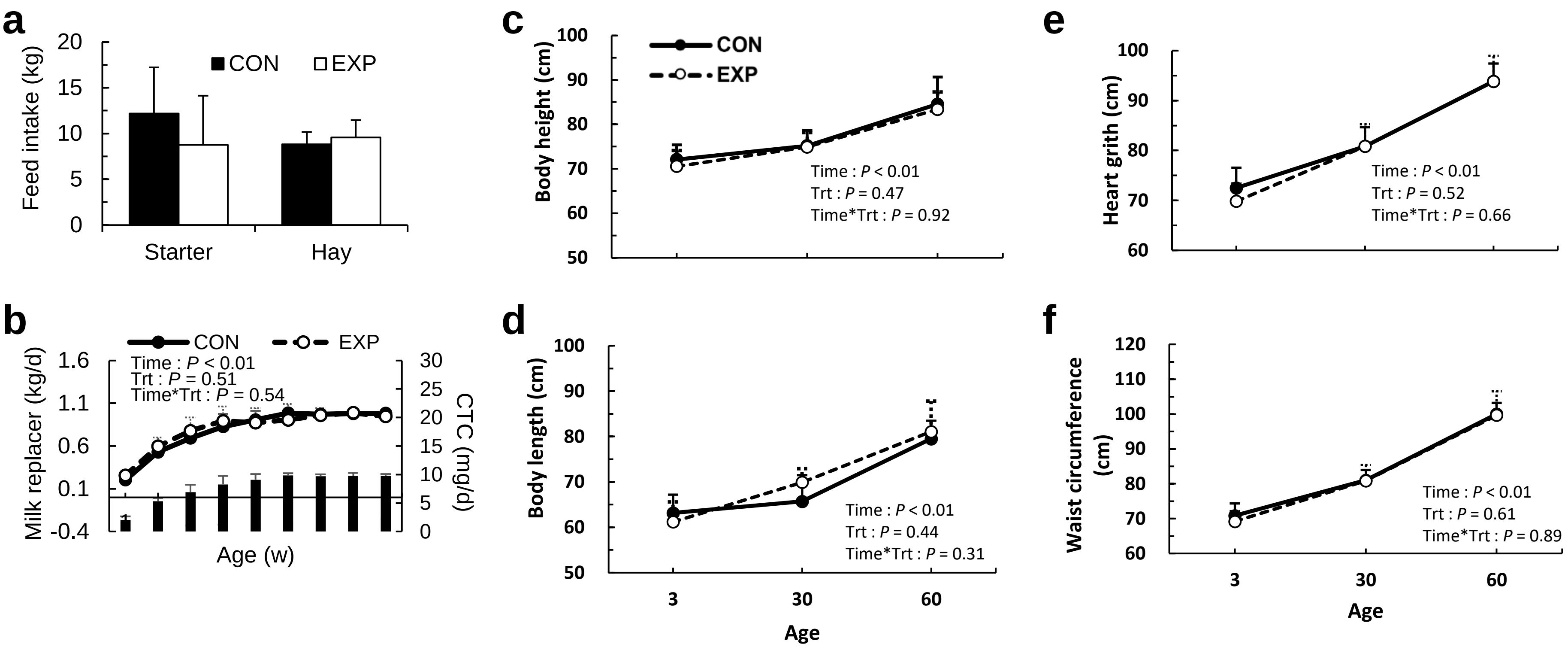

Figure S1. (a) feed intake and (b) antibiotic doses during the experimental period were shown, respectively. Doses of chlortetracycline (CTC) is expressed as bar graph. (d) shows changes in body weight during the period. Body height (c), body length (d), heart girth (e) and body waist circumference (f) at 3, 30, 60 days of age for the calves in fed milk replacer containing Chlortetracycline (CTC) at 10g/kg (CON) or 0g/kg (EXP). The values are means ± S.D.

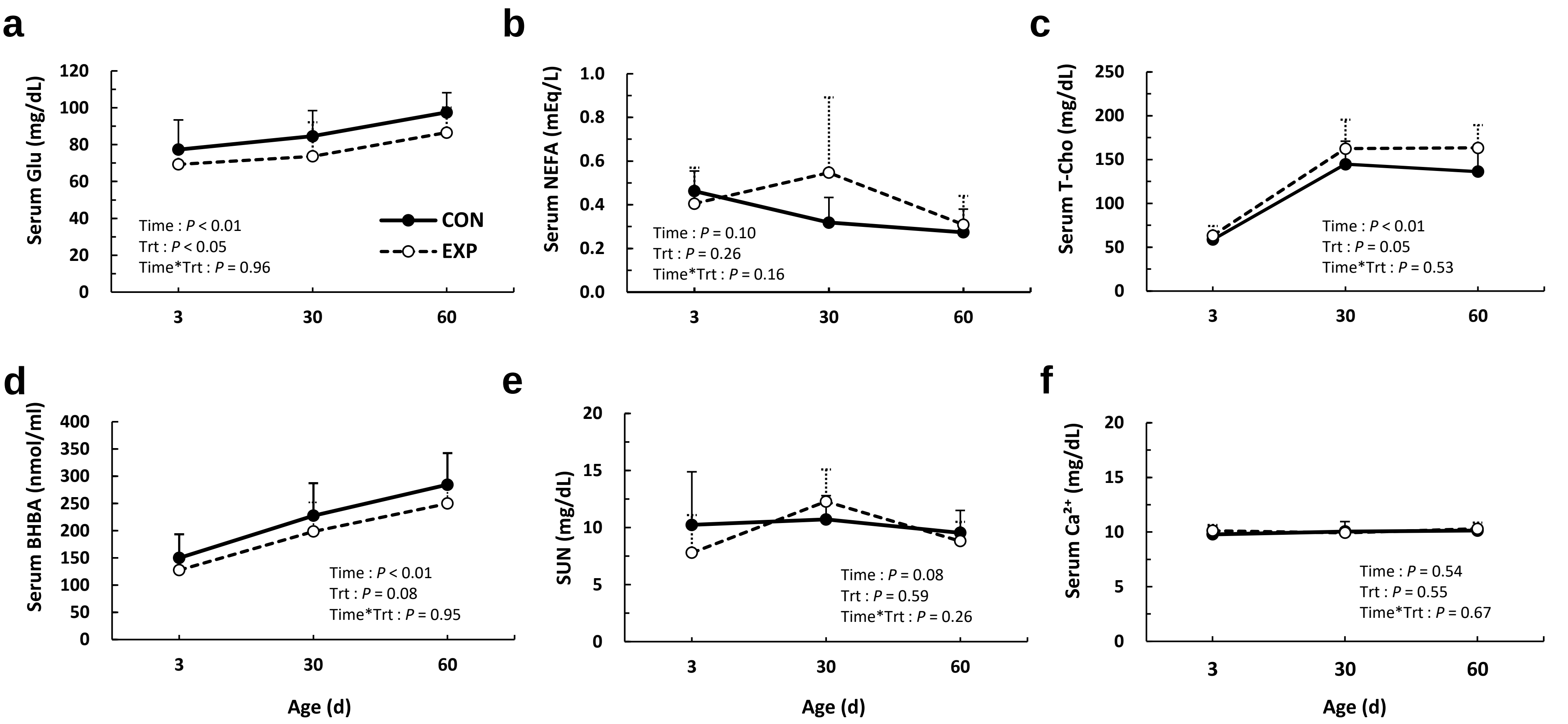

Figure S2. Blood components for calves fed milk replacer containing Chlortetracycline (CTC) at 10g/kg (CON) or 0g/kg (EXP). (a-f) Serum glucose (a), nonesterified fatty acid (NEFA) (b), total cholesterol (T-Cho) (c), β-hydroxybutyric acid (BHBA) (d), serum urea nitrogen (SUN) (e) and Ca^{2+} (f) concentrations for CON (closed circles and solid line) and EXP (open circles and dashed line). The values are means ± S.D.

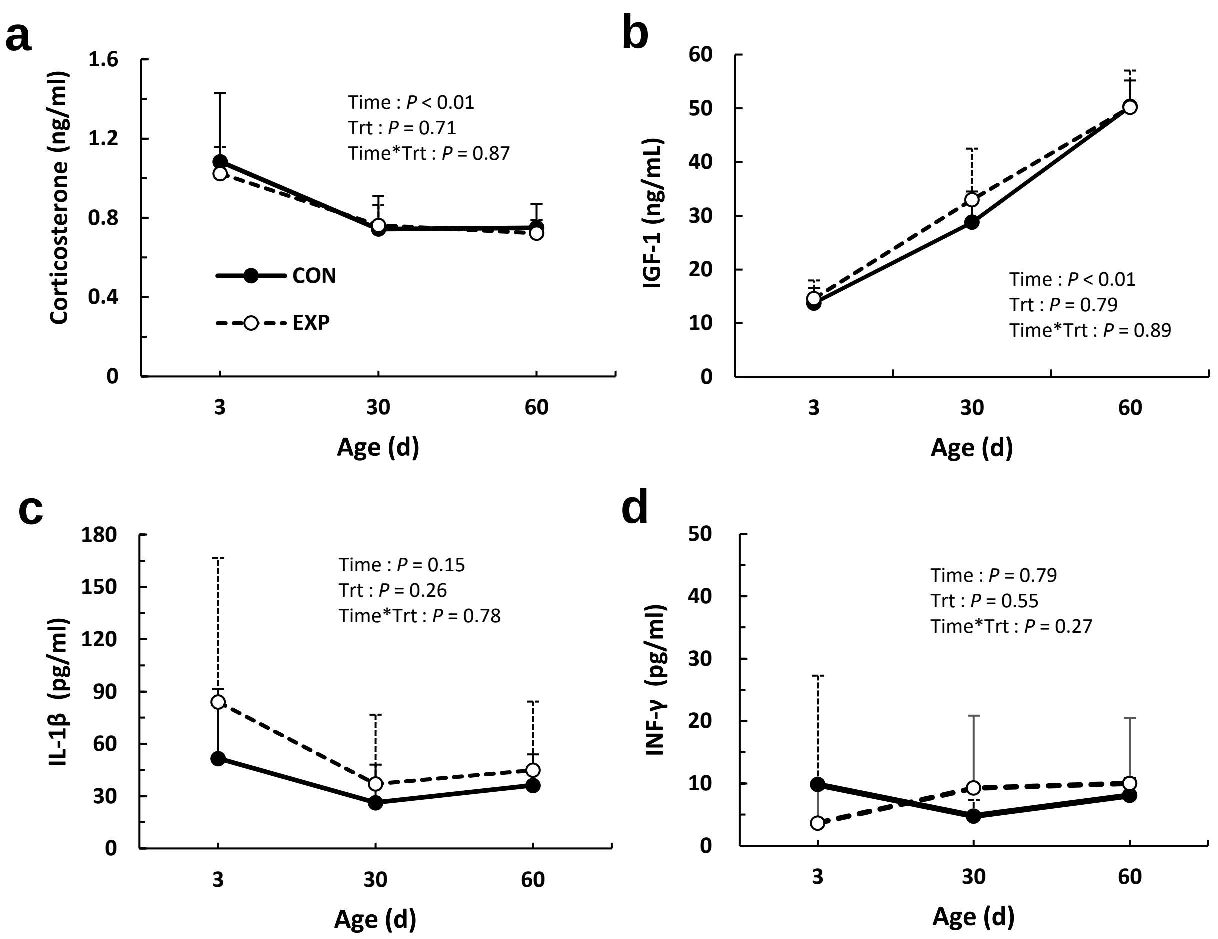

Figure S3. Blood components for calves fed milk replacer containing Chlortetracycline (CTC) (closed circles and solid line) at 10g/kg (CON) or 0g/kg (EXP) (open circles and dashed line). (a-d) Serum concentrations of corticosterone (a), insulin-like growth factor 1 (IGF-1) (b), interleukin-1 β (IL- β) (c), and interferon-γ (INF-γ). The values are means ± S.D.

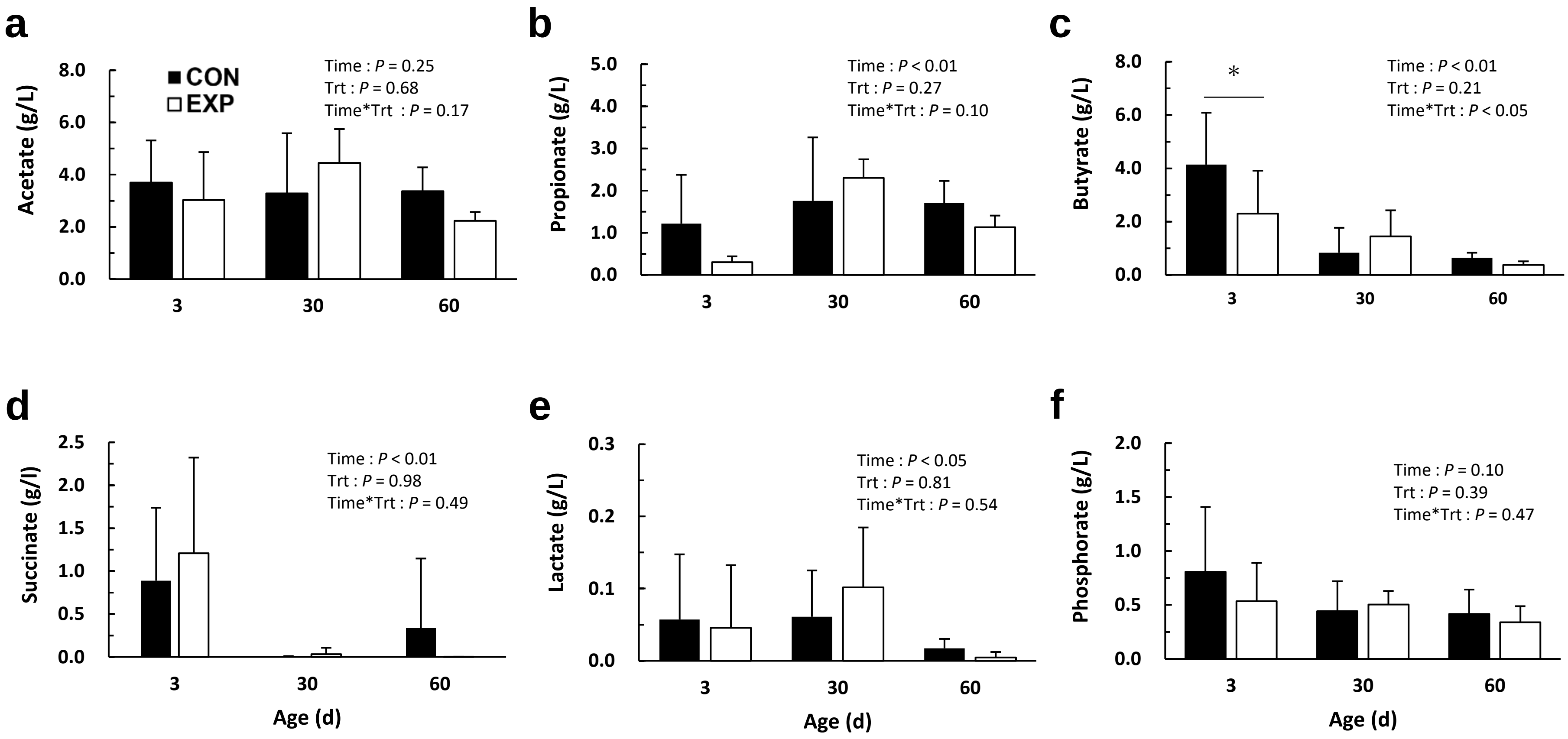

Figure S4. Fecal acetate (a), propionate (b), butyrate (c), succinate (d), lactate (e), and phosphate concentrations (f) at 3, 30, and 60 d of age for calves fed milk replacer containing Chlortetracycline (CTC) at 10g/kg (CON) or 0g/kg (EXP). The values are means ± S.D.

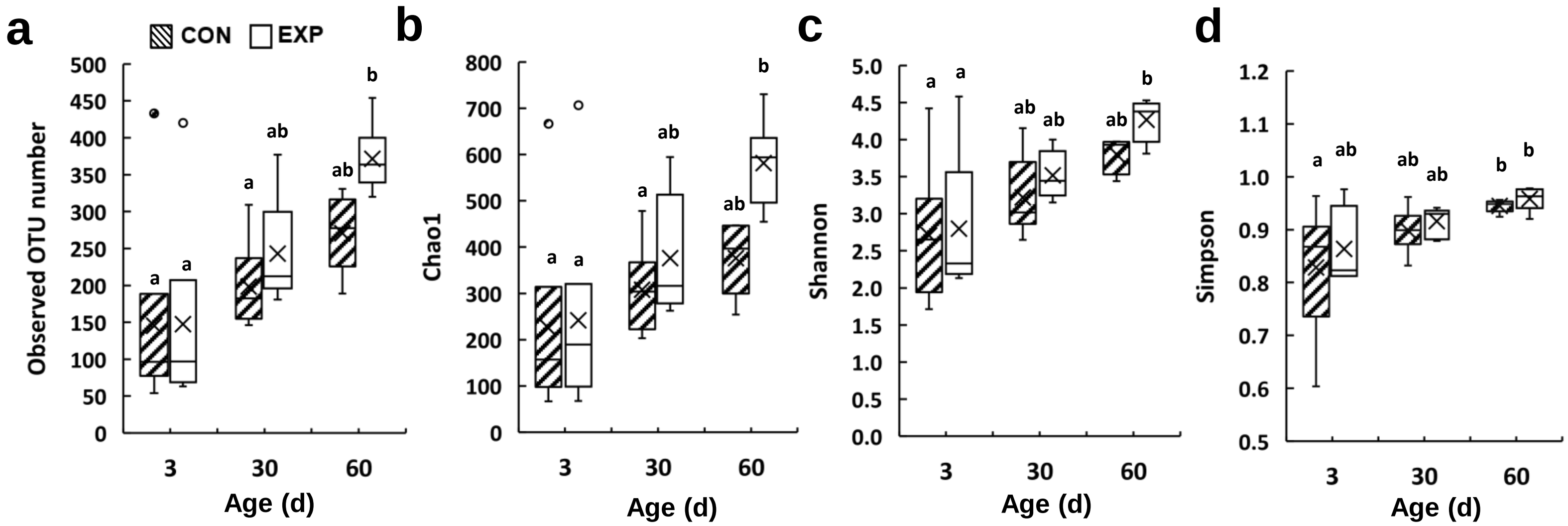

Figure S5. Alpha diversity indices including observed OTU number (a), Chao1 (b), Shannon (c) and Simpson (d) for the calves fed milk replacer containing Chlortetracycline (CTC) at 10g/kg (CON) or 0g/kg (EXP) at 3, 30 and 60 days of age. Each data was expressed as standard boxplots with medians and averages. Outliers are shown as dots. Different letters above boxplots within a treatment group show significant difference (Tukey's HSD $P < 0.05$). The values are means ± S.D.

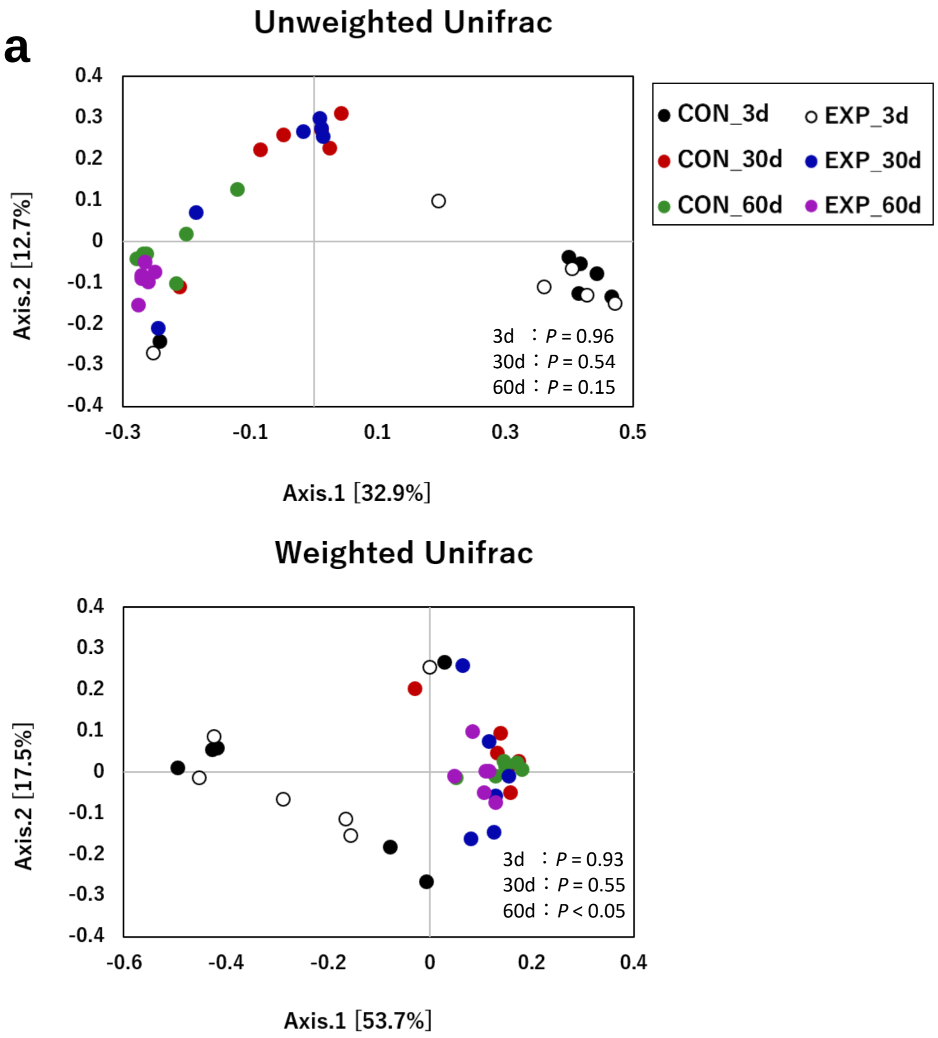

b

Category	vs	No. subject	Unweigthed UniFrac R^2	Unweigthed UniFrac *P* value	Weigthed UniFrac R^2	Weigthed UniFrac *P* value
CON	Day 3 vs Day 30	Day 3 : 6<br>Day 30 : 6	0.261	0.013	0.484	0.004
	Day 3 vs Day 60	Day 3 : 6<br>Day 60 : 6	0.328	0.009	0.361	0.007
	Day30 vs Day 60	Day 30 : 6<br>Day 30 : 6	0.222	0.011	0.106	0.356
EXT	Day 3 vs Day 30	Day 3 : 6<br>Day 30 : 6	0.246	0.015	0.462	0.001
	Day 3 vs Day 60	Day 3 : 6<br>Day 60 : 6	0.372	0.004	0.465	0.004
	Day30 vs Day 60	Day 30 : 6<br>Day 30 : 6	0.239	0.012	0.237	0.013
All	Day 3 vs Day 30	Day 3 : 6<br>Day 30 : 6	0.279	0.001	0.487	0.001
	Day 3 vs Day 60	Day 3 : 6<br>Day 60 : 6	0.362	0.001	0.414	0.001
	Day30 vs Day 60	Day 30 : 6<br>Day 30 : 6	0.275	0.002	0.214	0.032

Figure S6. Beta diversity index for the calves fed milk replacer containing Chlortetracycline (CTC) at 10g/kg (CON) or 0g/kg (EXP). (a) Indices for β-diversity were estimated using Unifrac analysis with weighted and unweighted principal coordinate analysis (PCoA). Results of Adonis test are shown as follows: Unweighted Unifrac 3d, $R^2 = 0.037$ and $P = 0.960$; 30d, $R^2=0.083$ and $P = 0.543$; 60d, $R^2 = 0.114$ and $P = 0.146$; Weighted Unifrac 3d, $R^2 = 0.030$ $P = 0.933$; 30d, $R^2 = 0.073$ and $P = 0.548$; and 60d, $R^2= 0.208$ and $P = 0.029$. (b) Temporal differences in unweighted- and weighted Unifrac distances among sampling points detected by Adonis. The values are means ± S.D.

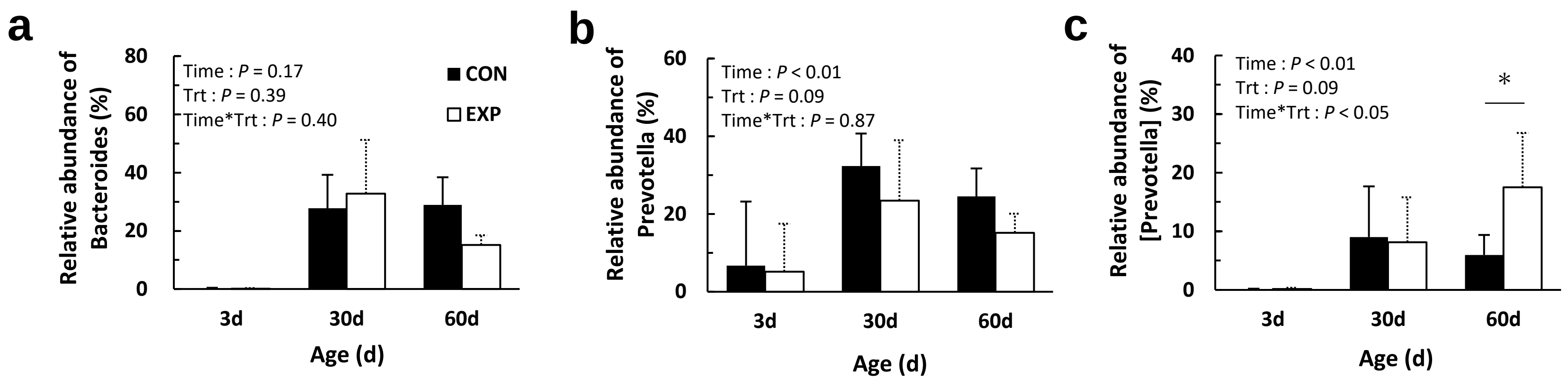

Figure S7. Relative abundance of (a) genus Bacteroides, (b) Prevotella, and (c) Prevotella-related bacteria for the CON and EXP.

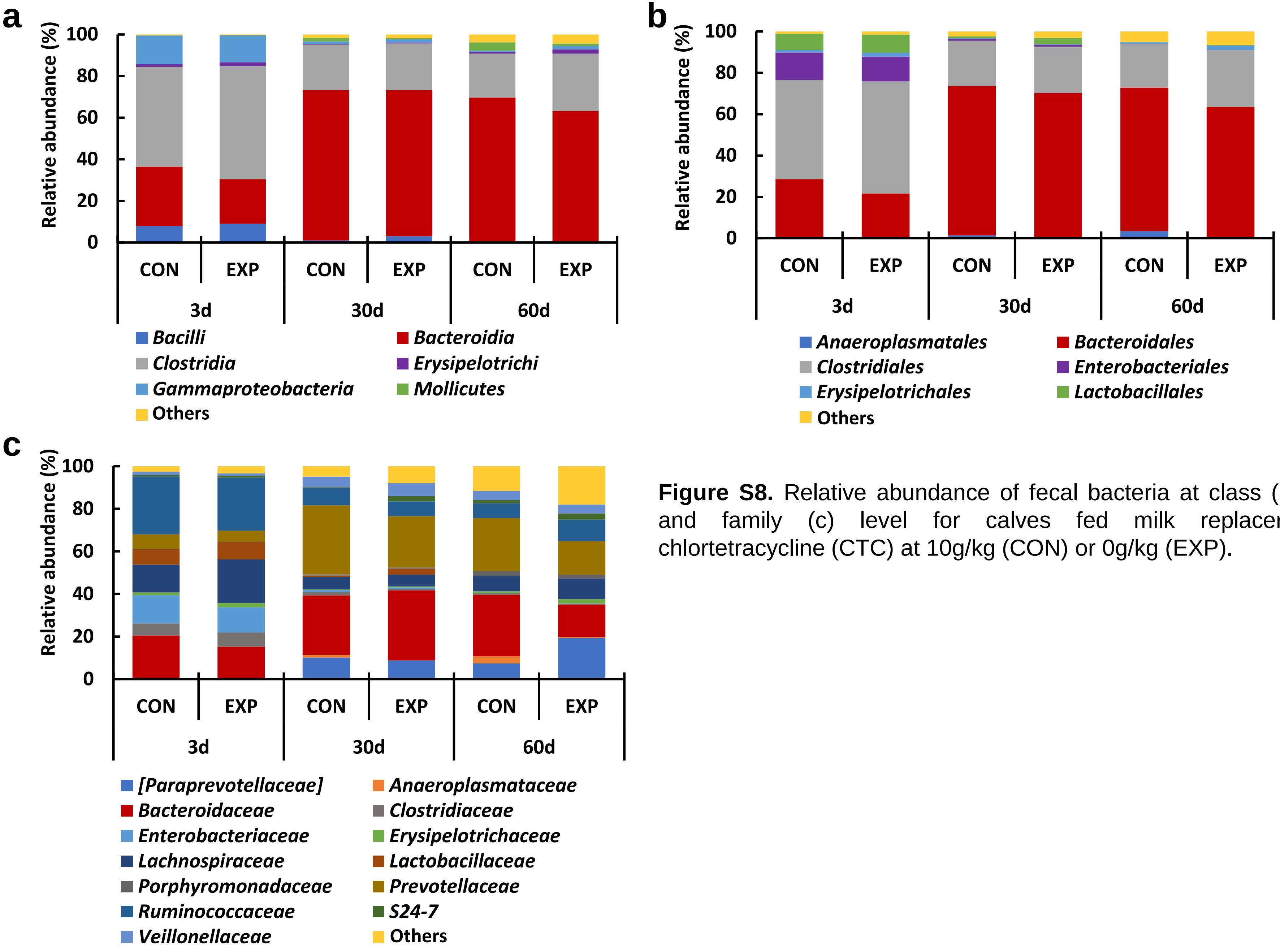

Figure S8. Relative abundance of fecal bacteria at class (a), order (b), and family (c) level for calves fed milk replacer containing chlortetracycline (CTC) at 10g/kg (CON) or 0g/kg (EXP).

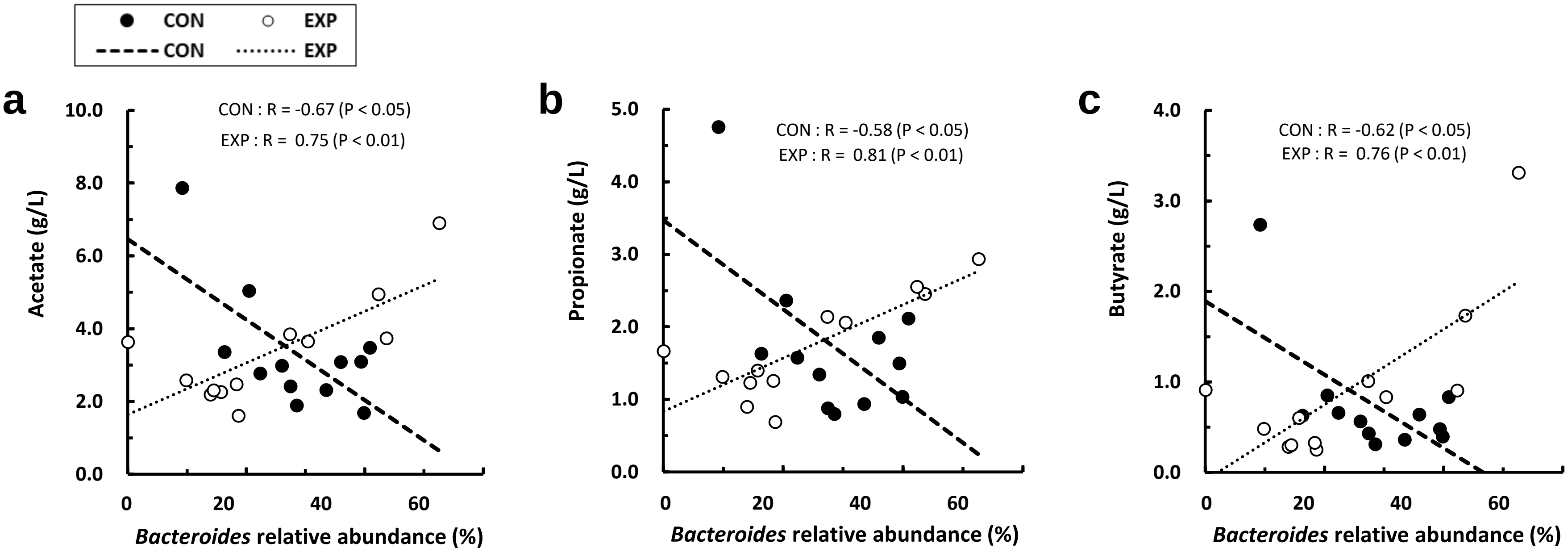

Figure S9. Correlation coefficient (r) between fecal abundance of genus Bacteroides and fecal concentrations of acetate (a), propionate (b) and butyrate (c).

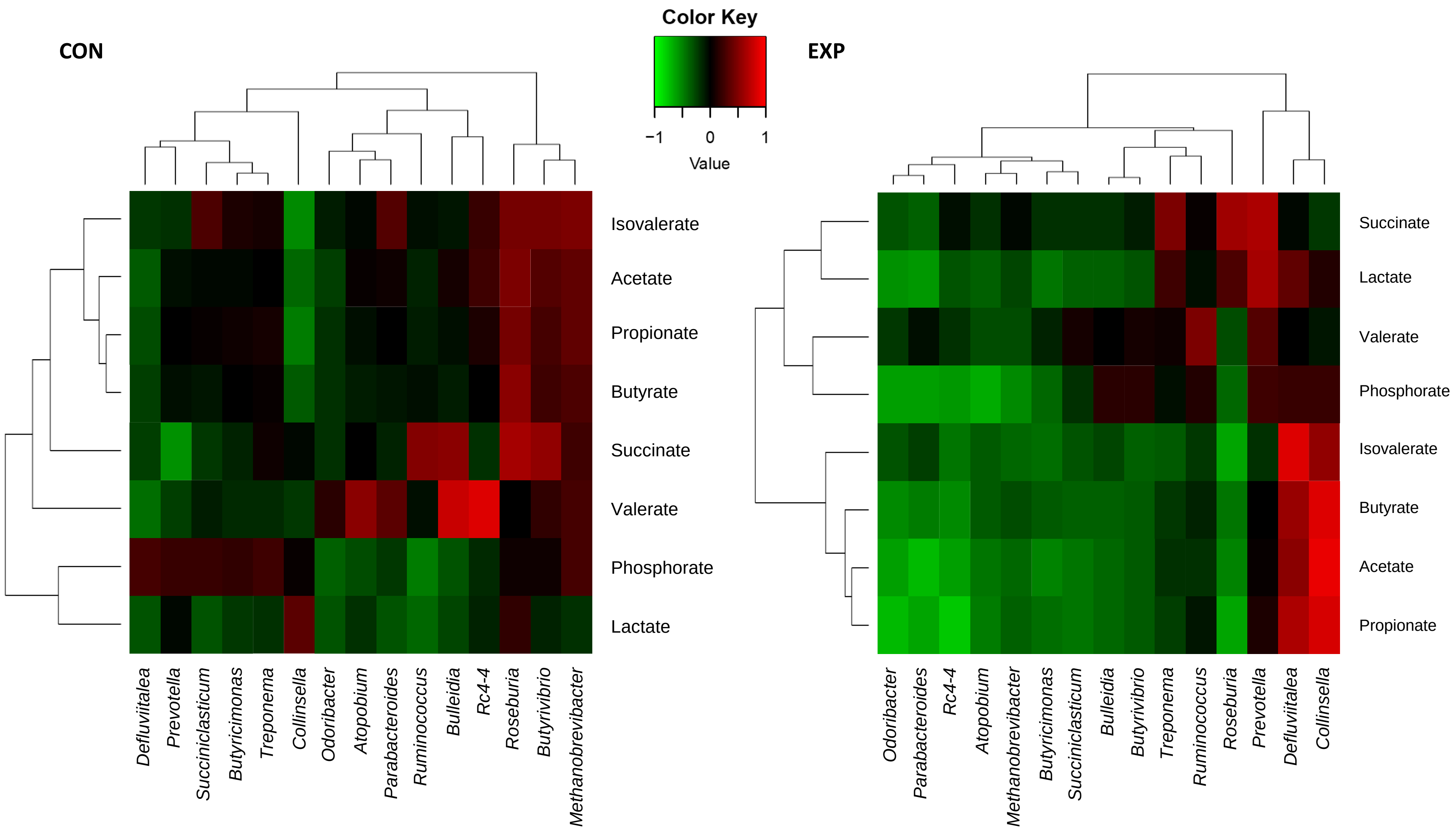

Figure S10. Heatmaps of correlations between the levels of short-chain fatty acids, lactate, succinate and phosphate and fecal bacterial abundance at the genus level selected by LDA. CON: the group treated with antibiotics; EXP: the group treated without antibiotics.

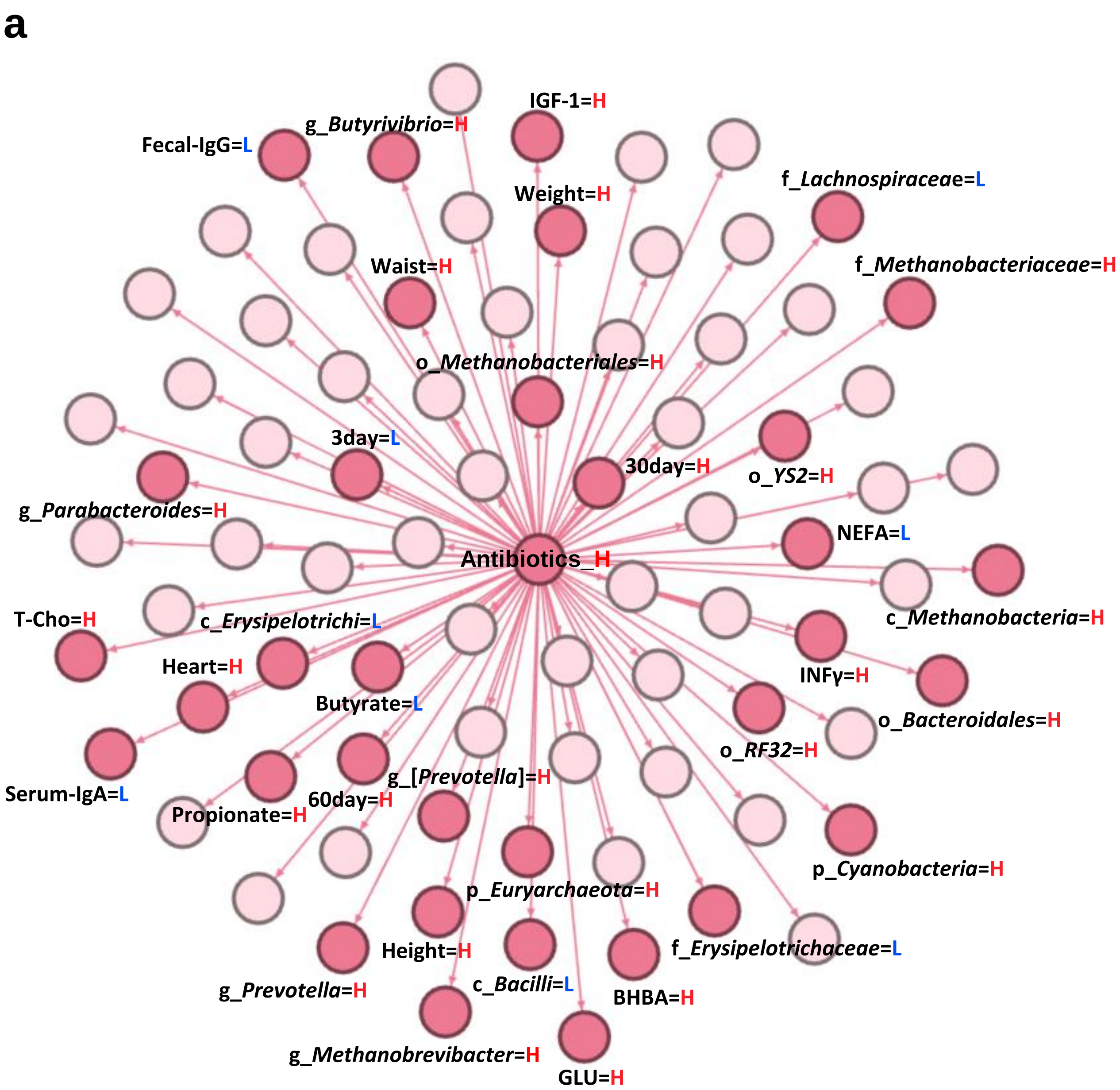

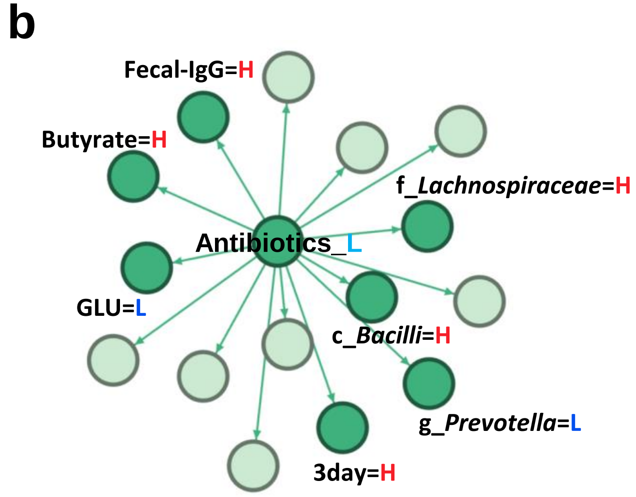

Figure S11. Association networks of factors sorted (a) with and (b) without antibiotic CTC treatment (> lift value 1.3 in the association analyses). All data are clearly stated in the supplementary data (the filename “Data S1 Finalnew.xlsx”). Positive and negative associated relationships are divided into high (_H) (red color) or low (_L) (blue color) levels based on the mediation values of the whole dataset of targeted components. Factors shown in darker color indicate bacterial groups detected in the LDA, as well as indicators of physiological and growth stages. The abbreviation show as follows: Antibiotics, CTC treatement; g_: genus; f_: family; o_: order; c_: class; p_: phylum; Heart: Heart girth_Physique; Height: Withers height_Physique; Waist: Waist circumference_Physique; Weight: Body weight_Physique; GLU: serum glucose; BHBA: β-hydroxybutyric acid; NEFAs: serum nonesterified free fatty acids; T-Cho: serum total cholesterol; IGF-1: Insulin-like growth factor 1; INFγ: interferon γ; IgA: immunoglobulin A; IgG: immunogloblin G.

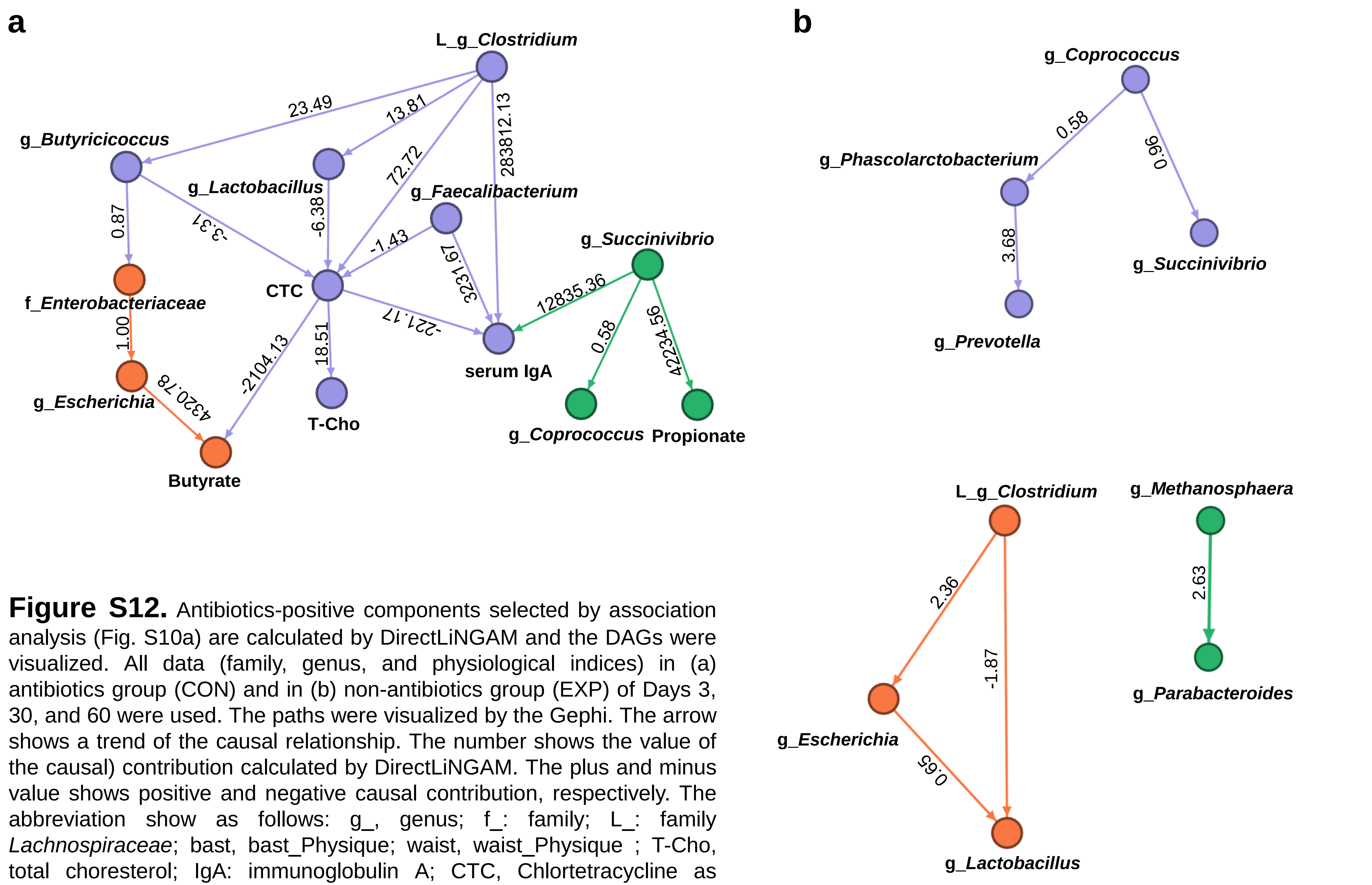

Figure S12. Antibiotics-positive components selected by association analysis (Fig. S10a) are calculated by DirectLiNGAM and the DAGs were visualized. All data (family, genus, and physiological indices) in (a) antibiotics group (CON) and in (b) non-antibiotics group (EXP) of Days 3, 30, and 60 were used. The paths were visualized by the Gephi. The arrow shows a trend of the causal relationship. The number shows the value of the causal) contribution calculated by DirectLiNGAM. The plus and minus value shows positive and negative causal contribution, respectively. The abbreviation show as follows: g_, genus; f_: family; L_: family *Lachnospiraceae*; bast, bast_Physique; waist, waist_Physique ; T-Cho, total choresterol; IgA: immunoglobulin A; CTC, Chlortetracycline as antibiotics.

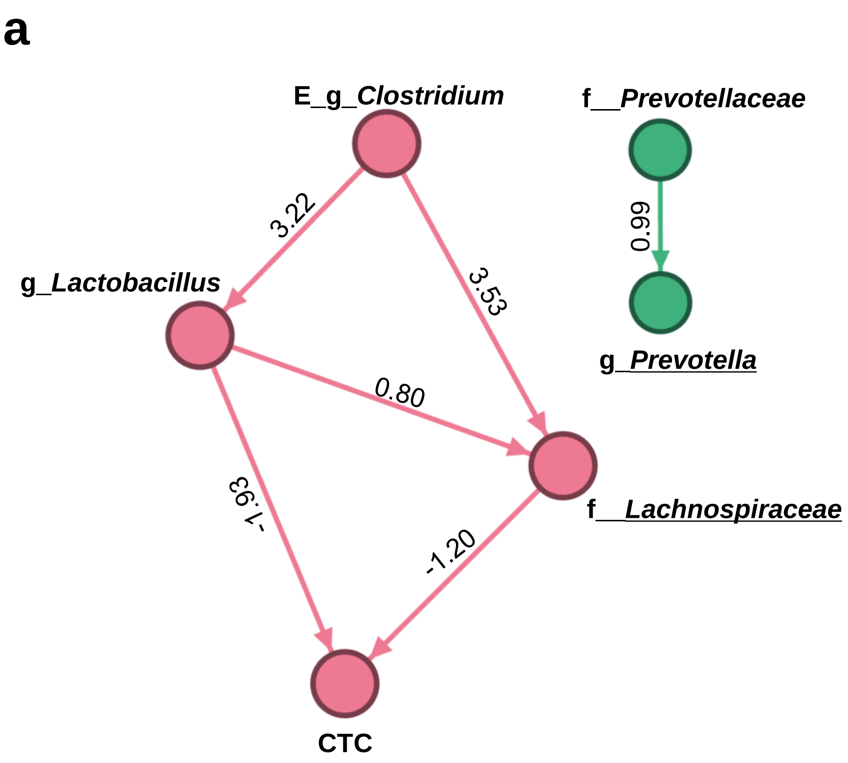

Figure S13. DirectLiNGAM-caluculated results for antibiotics-negative components (Fig. S10b) selected by association analysis are shown. All data (family and genus) in (a) antibiotics group (CON) and in (b) non-antibiotics group (EXP) of Days 3, 30, and 60 were used. The paths based on all the data were visualized by the Gephi, respectively. The arrow shows a trend of the causal relationship. The number shows the value of the causal contribution calculated by DirectLiNGAM. The plus and minus cost shows positive and negative causal contribution, respectively. The abbreviation show as follows: g_, genus; f_: family; E_: family *Erysipelotrichaceae;* CTC, Chlortetracycline as antibiotics.

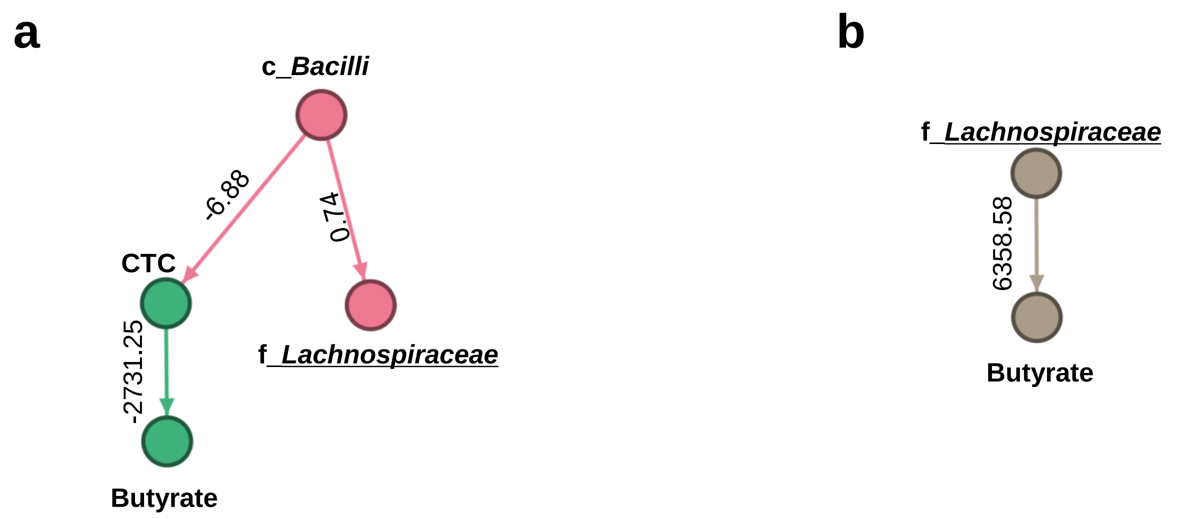

Figure S14. DirectLiNGAM-caluculated results of the components associated with class Bacilli (> lift value 1.3 in the association analyses) in group without antibiotics (Fig. S10b) are shown. All data (family and genus) in (a) antibiotics group (CON) and in (b) non-antibiotics group (EXP) of Days 3, 30, and 60 were used. The arrow shows a trend of the causal relationship. The number shows the value of the causal contribution calculated by DirectLiNGAM. The abbreviation show as follows: f_, family, c_ class; CTC, Chlortetracycline as antibiotics.
