# Supplementary material for: Estimation of silent phenotypes of calf antibiotic dysbiosis
Source: Sci Rep. 2023 Apr 19;13:6359. doi: 10.1038/s41598-023-33444-0 (PMC10115819; doi:10.1038/s41598-023-33444-0)
Supplement: Supplementary file 3 — Supplementary Information 3. [file 41598_2023_33444_MOESM3_ESM.docx]

**Supplementary Methods**

**Estimation of silent phenotypes of calf antibiotic dysbiosis**

*Shunnosuke Okada*^1^, *Yudai Inabu^1^, Hirokuni Miyamoto***^2,3,4,5^, Kenta Suzuki^6^, Tamotsu Kato^3^,*

*Atsushi Kurotani^7,8^, Yutaka Taguchi^1^, Ryoichi Fujino^1^, Yuji Shiotsuka^1^, Tetsuji Etoh^1^, Naoko Tsuji^5^,*

*Makiko Matsuura^2,5^, Arisa Tsuboi^,4,5,7^, Akira Saito^9^, Hiroshi Masuya^6^,*

*Jun Kikuchi^7^, Yuya Nagasawa^10^, Aya Hirose^10^,* *Tomohito Hayashi^10^, Hiroshi Ohno^3^***,*

*Hideyuki Takahashi^1^**

*Affiliations:*

*^1^Kuju Agricultural Research Center, Graduate School of Agriculture, Kyushu University, Oita, Japan, 878-0201*

*^2^Graduate School of Horticulture, Chiba University, Chiba, Japan, 263-8522*

*^3^RIKEN Integrated Medical Science Center, Yokohama, Kanagawa, Japan, 230-0045*

*^4^Japan Eco-science (Nikkan Kagaku) Co., Ltd., Chiba, Japan, 260-0034*

*^5^Sermas, Co., Ltd., Chiba, Japan, 271-8501*

*^6^RIKEN, BioResource Research Center, Tsukuba, Ibaraki, Japan, 305-0074*

*^7^RIKEN Center for Sustainable Resource Science, Yokohama, Kanagawa, Japan, 230-0045*

*^8^Research Center for Agricultural Information Technology, National Agriculture and Food Research Organization, Tsukuba, Ibaraki, Japan, 305-0856*

*^9^Feed‐Livestock and Guidance Department, Dairy Technology Research Institute, The National Federation of Dairy Co‐operative Associations (ZEN‐RAKU‐REN), Fukushima, Japan*

*^10^Pathology and Production Disease Group, Division of Hygiene Management, Hokkaido Research Station, National Institute of Animal Health, National Agriculture and Food Research Organization, Hokkaido, Japan, 062-0045.*

* Cocorrespondence:

Hirokuni Miyamoto Ph.D., hirokuni.miyamoto@riken.jp, h-miyamoto@faculty.chiba-u.jp

Hiroshi Ohno Ph.D. and M.D., RIKEN IMS, hiroshi.ohno@riken.jp

Hideyuki Takahashi Ph.D., [takahashi.hideyuki.990@m.kyushu-u.ac.jp](mailto:takahashi.hideyuki.990@m.kyushu-u.ac.jp)

**Appendix Explanation of Methods**

***Analyses of serum and fecal IgA, IgG and IFN-γ concentrations***

One hundred milligrams of feces were soaked in 1 ml PBS at room temperature for 1 h and centrifuged for 10 min at 10000 × g. After centrifugation, the supernatants were collected for fecal extraction. Total immunoglobulin A (IgA) and IgG concentrations in serum and fecal samples were determined by employing quantitative sandwich ELISA. Ninety-six microtiter plates (C96 Maxisorpcert, Nunc-Immuno Plate, Thermo Fisher Scientific) were directly coated overnight at 4 °C with 0.33 µg/well capture IgA antibody (A10-131A, Bethyl Laboratories, Inc., Montgomery, TX, USA) or 0.2 µg/well capture IgG antibody (A10-118A, Bethyl Laboratories Inc.). After incubation, wells were washed with Tris-buffered saline-Tween-20 (TBST) and then incubated with 100 μL of serum (IgA = diluted 1:10000, IgG = diluted 1:100000 in PBS) or feces (IgA = diluted 1:10, IgG = diluted 1:1 in PBS) for 90 min at room temperature. After five TBST washes, wells were incubated with horseradish peroxidase-conjugated sheep anti-bovine IgA antibody (diluted 1:30000, A10-131P, Bethyl Laboratories, Inc.) or horseradish peroxidase-conjugated sheep anti-bovine IgG antibody (diluted 1:100000, A10-118P, Bethyl Laboratories, Inc.) for 2 h at room temperature. Freshly prepared substrate was added, and the OD was measured at 450 nm using a 3′,3′,5,5′-tetramethylbenzidine microwell peroxidase substrate system (KPL, Gaithersburg, MD, USA) with a spectrophotometer (Multiskan GO, Thermo Fisher Scientific). All samples were analyzed in duplicate, and mean values were calculated. Final IgG and IgA concentrations (mg/mL) in serum and feces were calculated using the standard curve generated for each individual assay taking dilutions into account. Serum and fecal concentrations of interferon γ (IFN-γ) were measured using an ELISA kit (3119-1H-20, Mabtech, Nacka Strand, Sweden) according to the manufacturer’s instructions.

***Association analysis***

Association analysis, an elementary method of unsupervised learning used for market research and ecological analysis, is applied to achieve an understanding beyond the logic of numbers using relative numbers [1-4] as a suitable approach when the classification of data is different for each categorized layer. Here, all growth-related data, metabolomic data, and bacterial populations were analyzed and classified into associated components by subjecting them to conditions in which it is difficult to make horizontal comparisons.

In brief, effects from causes were classified as x and y, respectively. The probability (P) is defined as follows: support, (x ⇒ y) = P(x ∩ y); confidence, (x ⇒ y) = P(x ∩ y)/P(x); lift, (x ⇒ y) = P(x ∩ y)/P(x)P(y). A value of > 1 represents a positive association (if the value indicates independence), a value of < 1 represents a negative association.

Here, association rules were determined by using criterion values of support, confidence, and lift (“support = 0.2, confidence = 0.4, maxlen = 2” and “lift > 1.3”). The data combined with all information, such as body conditional and physiological data, fecal metabolites, and bacterial taxa obtained with or without antibiotic treatment (set as 1 or 0), were used in the analysis. To avoid the differences dependent upon the layers, all the data for the analysis were calculated for binarization based on the median value (M) within the data and sorted as 0 (< M) and 1 (> M). The packages “arules” and “aruleViz” in R software (https://cran.r-project.org) were applied. The association systemic network was rendered by Force Atlas with Noverlap in Gephi 0.9.2 (<https://gephi.org>).

***ELA interaction network***

The procedure iterates the following steps:

1. Set $t=0$, initial learning rate $\alpha_{0}=0.1$, logistic priors as $p_{h}=-tanh(h/2/2)/2$, $p_{g}=-tanh(g/2/2)/2$ and $p_{J}=-tanh(J/0.5/2)/2$ and initialize parameter values for $h, J, g$, and the expected sample states $X^{*}\left( 0 \right)=X$. We set $Y^{*}=Y$ throughout the calculation.

2. Calculate learning rate $\alpha$ as follows:

$$\alpha=\alpha_{0}\frac{5000}{4999+t},$$

momentum $m$ as follows:

$$m=0.9\left( 1-\frac{1}{0.1t+2} \right).$$

3. For $x_{i}^{*}(t)$ from $i=1$ to $N$, run one step heat-bath algorithm based on current parameters ($h$, $J$ and $g$): transition from the current community composition $\sigma^{\left( k \right)}$ to one of its $S$ adjacent community composition $\sigma^{\left( k^{'} \right)}$, selected with probability $1/S$, was attempted ($\sigma^{\left( k \right)}$ and $\sigma^{\left( k^{'} \right)}$ differs only with respect to the presence/absence status of one of *S* species). The transition to the selected state took place with probability $e^{-E\left( \sigma^{\left( k^{'} \right)}|ℇ^{(i)} \right)}/(e^{-E(\sigma^{\left( k \right)}|ℇ^{(i)})}+e^{-E(\sigma^{\left( k^{'} \right)|ℇ^{(i)}})})$. Here, ($e^{-E(\sigma^{\left( k \right)}|ℇ^{(i)})}$ and $e^{-E(\sigma^{\left( k^{'} \right)}|ℇ^{(i)})}$ are given by eq. (4). If transition occurs, the sample state is updated as $x_{i}^{*}(t)\leftarrow\sigma^{\left( k^{'} \right)}$.

4. Subtract the simulated sufficient statistics from the observed ones to calculate the approximate likelihood gradient. Sufficient statistics are calculated as, $SS_{1}^{*}=X^{*}{(X^{*})}^{t}$ (here, ${(X^{*})}^{t}$ is the transpose of $X^{*}$), and $\mathrm{SS}_{2}^{*}={(X^{*})}^{t}Y^{*}$. Then, we obtain the difference of sufficient statistics as:

$$\Delta SS_{1}=\mathrm{SS}_{1}-\mathrm{SS}_{1}^{*},$$

and

$$\Delta SS_{2}=\mathrm{SS}_{2}-\mathrm{SS}_{2}^{*}.$$

Here, $\mathrm{SS}_{1}$ and $\mathrm{SS}_{2}$ is the corresponding sufficient statistics calculated from actual data (i.e., $SS_{1}=XX^{t}$ and $SS_{2}=X^{t}Y$).

5. Adjust the model parameters to climb the approximate gradient, using a schedule of step sizes as:

$$h_{\mathrm{new}}\leftarrow h_{\mathrm{old}}+\Delta h_{\mathrm{new}},$$

$$J_{\mathrm{new}}\leftarrow J_{\mathrm{old}}+\Delta J_{\mathrm{new}},$$

$$g_{\mathrm{new}}\leftarrow g_{\mathrm{old}}+\Delta g_{\mathrm{new}}.$$

Here,

$$\Delta h_{\mathrm{new}}=\alpha G_{h} +m\Delta h_{\mathrm{old}},$$

$$\Delta J_{\mathrm{new}}=\alpha G_{J}+m\Delta J_{\mathrm{old}},$$

$$\Delta g_{\mathrm{new}}=\alpha G_{g}+m\Delta g_{\mathrm{old}},$$

and,

$$G_{h}=\frac{diag(\Delta\mathrm{SS}_{1})+p_{h}}{N},$$

$$G_{J}=\frac{\Delta\mathrm{SS}_{1}+p_{J}}{N}\left| I\left( S \right)-1 \right|,$$

$$G_{g}=\frac{\Delta\mathrm{SS}_{2}+p_{g}}{N},$$

are the approximated likelihood gradients. Here, $I\left( S \right)$ is a $S\times S$ identity matrix.

6. Set $h_{\mathrm{new}}$, $J_{\mathrm{new}}$, $g_{\mathrm{new}}$, $\Delta h_{\mathrm{new}}$, $\Delta J_{\mathrm{new}}$ and $\Delta g_{\mathrm{new}}$ as $h_{\mathrm{old}}$, $J_{\mathrm{old}}$, $g_{\mathrm{old}}$, $\Delta h_{\mathrm{old}}$, $\Delta J_{\mathrm{old}}$ and $\Delta g_{\mathrm{old}}$, respectively. If $t<T$, increment t by 1 and back to 2, else terminate the loop.

The simulations in Step 3 use one step heat-bath algorithm (Gibbs sampling) to generate a community composition distribution based on the model’s current parameter estimates. The approximate likelihood gradients in Step 5 match those of gradient descent, except that they are averaged over a set of Monte Carlo samples rather than over all possible community compositions. These gradients were augmented with a momentum term and regularizers based on a logistic prior with location 0 and scale 2.0 (for environmental responses) or 0.5 (for pairwise relationships). We set hyperparameters in this algorithm, including a maximum number of iteration steps $T=50000$, according to the preliminary analysis where we checked the convergence of model parameters.

**Supplementary Text**

***Analyses based on Data S1***

All raw data are summarized in the sheet name “whole raw data” of the Data S file (Excel filename: DataS1 Finalnew.xlsx). Based on these data, the optimized data for figure creation are shown as the sheet name as follows: Figs. 3 (data for LDA analysis), 4 (data for ELA) and 5 (data for ELA network and DirectLiNGAM); Figs. S1a (Total monthly feed intake), S1b (Milk replacer intake), S11 (raw data for association analysis), S11a (CON data calculated by association analysis), S11b (EXP raw data calculated by association analysis), S12a (raw data to analyze CON group by DirectLiNGAM analysis with components selected from Fig. S11a), S12b (raw data to analyze EXP group by DirectLiNGAM analysis with components selected from Fig. S11a), S13a (raw data to analyze CON group by DirectLiNGAM analysis with components selected from Fig. S11b), and S13b (raw data to analyze EXP group by DirectLiNGAM analysis with components selected from Fig. S11b), respectively.

**References**

[1] Shiokawa, Y., Misawa, T., Date, Y. & Kikuchi, J. Application of Market Basket Analysis for the Visualization of Transaction Data Based on Human Lifestyle and Spectroscopic Measurements. *Analytical Chemistry* **88**, 2714-2719 (2016).

[2] Shiokawa, Y., Date, Y. & Kikuchi, J. Application of kernel principal component analysis and computational machine learning to exploration of metabolites strongly associated with diet. *Sci Rep* **8**, 3426 (2018).

[3] Wei, F., Sakata, K., Asakura, T., Date, Y. & Kikuchi, J. Systemic Homeostasis in Metabolome, Ionome, and Microbiome of Wild Yellowfin Goby in Estuarine Ecosystem. *Sci Rep* **8**, 3478 (2018).

[4] Miyamoto, H. et al. A potential network structure of symbiotic bacteria involved in carbon and nitrogen metabolism of wood-utilizing insect larvae. *Sci Total Environ* **836**, 155520 (2022).
